# Characterization of clumpy adhesion of *Escherichia coli* to human cells and associated factors influencing antibiotic sensitivity

**DOI:** 10.1128/spectrum.02606-23

**Published:** 2024-03-26

**Authors:** Muhammad Moman Khan, Katarzyna Sidorczuk, Juliane Becker, Adrianna Aleksandrowicz, Karolina Baraniewicz, Christina Ludwig, Aamir Ali, Robert A. Kingsley, Peter Schierack, Rafał Kolenda

**Affiliations:** 1 Institute for Biotechnology, Brandenburg University of Technology (BTU) Cottbus-Senftenberg, Senftenberg, Germany; 2 Department of Bioinformatics and Genomics, Faculty of Biotechnology, University of Wrocław, Wrocław, Poland; 3 Department of Biochemistry and Molecular Biology, Faculty of Veterinary Medicine, Wrocław University of Environmental and Life Sciences, Wrocław, Poland; 4 Department of Microbiology, Institute of Genetics and Microbiology, University of Wrocław, Wrocław, Poland; 5 Bavarian Center for Biomolecular Mass Spectrometry (BayBioMS), School of Life Sciences, Technical University Munich (TUM), Munich, Germany; 6 National Institute for Biotechnology and Genetic Engineering College, Pakistan Institute of Engineering and Applied Sciences (NIBGE-C, PIEAS), Faisalabad, Pakistan; 7 Quadram Institute Biosciences, Norwich Research Park, Norwich, United Kingdom; Institute of Parasitology, Biology Centre, ASCR, Ceske Budejovice, Czechia

**Keywords:** *Escherichia coli*, clumpy adhesion, motility, antibiotic tolerance, stress, adhesion

## Abstract

*Escherichia coli* intestinal infection pathotypes are characterized by distinct adhesion patterns, including the recently described clumpy adhesion phenotype. Here, we identify and characterize the genetic factors contributing to the clumpy adhesion of *E. coli* strain 4972. In this strain, the transcriptome and proteome of adhered bacteria were found to be distinct from planktonic bacteria in the supernatant. A total of 622 genes in the transcriptome were differentially expressed in bacteria present in clumps relative to the planktonic bacteria. Seven genes targeted for disruption had variable distribution in different pathotypes and nonpathogenic *E. coli,* with the *pilV* and *spnT* genes being the least frequent or absent from most groups. Deletion (Δ) of five differentially expressed genes, *flgH*, *ffp*, *pilV*, *spnT,* and *yggT,* affected motility, adhesion, or antibiotic stress. Δ*flgH* exhibited 80% decrease and Δ*yggT* depicted 184% increase in adhesion, and upon complementation, adhesion was significantly reduced to 13%. Δ*flgH* lost motility and was regenerated when complemented, whereas Δ*ffp* had significantly increased motility, and reintroduction of the same gene reduced it to the wild-type level. The clumps produced by Δ*ffp* and Δ*spnT* were more resistant and protected the bacteria, with Δ*spnT* showing the best clump formation in terms of ampicillin stress protection. Δ*yggT* had the lowest tolerance to gentamicin, where the antibiotic stress completely eliminated the bacteria. Overall, we were able to investigate the influence of clump formation on cell surface adhesion and antimicrobial tolerance, with the contribution of several factors crucial to clump formation on susceptibility to the selected antibiotics.

**IMPORTANCE:**

The study explores a biofilm-like clumpy adhesion phenotype in *Escherichia coli,* along with various factors and implications for antibiotic susceptibility. The phenotype permitted the bacteria to survive the onslaught of high antibiotic concentrations. Profiles of the transcriptome and proteome allowed the differentiation between adhered bacteria in clumps and planktonic bacteria in the supernatant. The deletion mutants of genes differentially expressed between adhered and planktonic bacteria, i.e., *flgH*, *ffp*, *pilV*, *spnT*, and *yggT,* and respective complementations in *trans* cemented their roles in multiple capacities. *ffp*, an uncharacterized gene, is involved in motility and resistance to ampicillin in a clumpy state. The work also affirms for the first time the role of the *yggT* gene in adhesion and its involvement in susceptibility against another aminoglycoside antibiotic, i.e., gentamicin. Overall, the study contributes to the mechanisms of biofilm-like adhesion phenotype and understanding of the antimicrobial therapy failures and infections of *E. coli*.

## INTRODUCTION

*Escherichia coli* is present in the gut microbiota of animals soon after birth, contributing to colonization resistance to pathogens, but it may also cause diseases that have the potential to move across and affect multiple hosts and be fatal (1). Disseminated infections may require treatment with antibiotics and although *E. coli* is inherently sensitive to almost all classes of antibiotics, frequent acquisition of resistance genes through horizontal gene transfer complicates treatment and contributes to resistance in other pathogens (2). *E. coli* is the leading cause of death from bacterial infections, attributable to and associated with antimicrobial resistance, totaling over 800,000 per year (3). *E. coli* is a priority pathogen included in the list of multidrug-resistant bacteria issued by the World Health Organization, which needs to be addressed to counter this global public health challenge (4).

Colonization by *E. coli* pathotypes is initiated by contact with the host gut mucosal epithelium, and adherence is important in overcoming mechanical removal and clearance of pathogens from the gut (5). Extracellular appendages and adhesins play a critical role in the specific binding of the bacterial cell to receptors on the host cell (6). Adhesion also facilitates the deployment of virulence factors that may include the transfer of effector proteins into host enterocytes through type III secretion systems. Diverse mechanisms of colonization and infection result in a variety of adhesion patterns that are associated with specific *E. coli* pathotypes. Enteroaggregative *E. coli* (EAEC) display an aggregative adherence pattern or localized adhesion due to the autoagglutination of bacterial cells shown by the stacked-brick configuration, which occurs on the host cell surface usually in the presence of D-mannose. Similarly, enteropathogenic *E. coli* (EPEC) also portrayed an aggregative adherence pattern mediated by specific fimbriae encoded on plasmids. A specialized adherence mechanism known as the attaching and effacing lesion involves intimate attachment to the host surface, followed by effacement of microvilli and cytoskeletal rearrangements. Diffusely adherent *E. coli* (DAEC) are characterized by scattered distribution over the host cell surface, and the adhesion pattern is referred to as diffuse adherence (7, 8). The importance of adhesion patterns in pathogenesis is not well understood, but they may also affect the sensitivity to antimicrobial agents or other environmental stressors present in the intestine due to decreased penetration of the stressor, in contrast to diffusely distributed single bacteria (9).

In a study of 282 *E. coli* strains isolated from diverse host species, four distinct adhesion patterns on a variety of cell types and tissues were observed: (i) diffusely distributed single bacteria; (ii) microcolonies; (iii) chains; and (iv) clumps (10). Adhesion as clumps was rare, only observed for two *E. coli* strains, and more pronounced on the human urinary bladder cell line 5637 (10). Clumps of bacteria were large, three-dimensional structures similar to those of biofilms and composed of several thousand bacteria that formed after only 4 h of incubation. Clumps constituted larger and more complex structures covering the cell line extensively and were resistant to repeated washing, whereas microcolonies formed simpler aggregates of clustered bacteria when visualized under the fluorescent microscope. Little is known about the bacterial factors required for clump formation and its impact on resistance to stressors. We, therefore, tested the hypothesis that bacteria present in *E. coli* strain 4972 clumps exhibit distinct gene expression profiles and that some genes with altered expression are involved in clumpy adhesion. We also investigated the contribution of *E. coli* strain 4972-encoded factors that affected clump formation on the susceptibility to the antibiotics.

## MATERIALS AND METHODS

### Bacterial isolates and plasmids

The strains and plasmids used in this study are listed in Table S1.

### Next-generation sequencing and genome analysis

Genomic DNA of *E. coli* strain 4972 was extracted using Wizard Genomic DNA Purification Kit (Promega Corp., Madison, USA) from 5 mL overnight culture as per the manufacturer’s instructions. The extracted nucleic acid was sequenced (Illumina), assembled with Shovill (11), and annotated using PROKKA (12) software. The whole genome sequence of strain 4972 is freely available from the GenBank database under accession number GCA_018429805.1 (13). To compare the phylogenetic relationship of strain 4972 in the context of previously published *E. coli* genomes, a similar approach described previously was selected (14). Genome sequences of *E. coli* genomes were downloaded from NCBI GenBank using accession numbers (Table S2), and SNPs in the core genome alignment were identified using snippy software, with reference to *E. coli* K-12 MG1655 (GenBank accession no.: CP005930). A phylogenetic tree was constructed using RAxML (15). Phylogroup and pathotype information for reference strains (Table S2) was described previously (14) and annotated on the tree using ggtree R package (16).

### Adhesion assays

The cell line of a urinary bladder carcinoma—5637, was cultivated in RPMI medium with the addition of 10% fetal bovine serum, 2 mM L-glutamine, 1 mM sodium pyruvate, and 1% penicillin/streptomycin. For the assay, a medium without antibiotics was used (later referred to as infection medium). Cells were grown at 37°C, 95% humidity, and 5% CO_2_ and passaged when the flask was of 80% ± 10% confluent. To visualize and semi-quantify the bacterial clumps, an adhesion assay was conducted in a 96-well format where the wild-type strain 4972, 4972 transformed with pFPV25.1Kan (encoding GFP), and deletion mutants were allowed to adhere to the monolayer of 5637 cells for 4 h, stained with propidium iodide (PI) or DAPI (4′,6-diamidino-2-phenylindole), and visualized using automated fluorescence microscopy-based Aklides system according to the already published methodology (10).

As it was not possible to fully quantify the clumpy adhesion phenotype of the mutants with respect to the wild type, quantification of colony-forming units (CFU) was done. For this, 5637 cells were seeded onto 24-well plates, and the assay was conducted when a confluent monolayer had formed. Prior to the addition of bacterial strains, monolayers of 5637 were washed with PBS and then 500 µL of infection medium was pipetted in each well. The day before reaching the monolayer, chosen bacteria were inoculated in 1 mL of LB and incubated at 180 rpm at 37°C for 16 h. The next day, OD_600_ of the cultures was measured and diluted to OD_600_ = 0.05 in 5 mL LB medium and grown at 220 rpm, 37°C to OD_600_ = 2.0. If a mutant with a pBAD33 expression vector was used, L-arabinose was added with a final concentration of 0.2% after reaching OD_600_ = 0.5, and bacteria were allowed to grow for another 2 h. After that, 1 mL of bacterial culture was washed with PBS, resuspended in 1 mL of infection medium, and bacterial OD_600_ was determined. For each strain, 300 µL of infection medium containing 1.2 × 10^5^ bacteria was added to each of the four well replicates. The plates were incubated at 37°C for 4 h, and each well was washed three times with 1 mL of PBS. Next, 1 mL of PBS with 1% Triton X-100 was added per well, the plate was incubated for 5 min at room temperature, and finally, the contents of the well were scraped by pipetting in each well and mixed thoroughly. A volume of 200 µL from each well was then transferred into 96-well plates and a dilution series was generated. On LB agar plates, 20 µL spots of the dilutions were pipetted in triplicates for each well. The plates were placed in the incubator for overnight growth at 37°C, and bacterial colonies were counted the next morning.

Bacteria within biofilm-like clumps were stressed using a modification of the adhesion assay in 24-well plates. Initially, dose-ranging experiments for ampicillin and gentamicin were carried out by a gradual increase in concentration in the infection medium after the clump formation of the wild-type strain. For ampicillin, the concentration tested for stress ranged from 0 to 16,000 µg/mL, whereas for gentamicin 0–16 µg/mL were tested. The concentration for exposure to each antibiotic, which resulted in a decrease in more than half of the viable bacteria, was selected. After the 4-h incubation and clump formation, the infection media were aspirated, and a fresh infection medium containing the antibiotics was added. The plate was incubated for another 1 h at 37°C. The rest of the procedure was identical to that for the adhesion assay.

### Biofilm assay

The strain 4972 was tested for biofilm formation using VideoScan in a variety of media: LB, LB supplemented with glucose (LB+), tryptic soy broth (TSB), TSB supplemented with glucose (TSB+), brain heart infusion (BHI) media, BHI supplemented with glucose (BHI+), M63 minimal media, M63 minimal media supplemented with glucose (M63+), RPMI cell culture media, and DMEM cell culture media using the already established protocol (17).

### RNA isolation from *E. coli* strain 4972 and sequencing

Adhesion assay on three different cell lines, i.e., porcine kidney (PK15), human intestinal (Caco-2), and urinary bladder carcinoma 5637, was carried out, where strain 4972 was allowed to adhere to these cell lines for 4 h with their respective mediums as detailed in Table S3. The procedure was the same for each cell line, whereby unattached bacteria in the supernatant were collected, the adhered bacterial cells were carefully removed by pipetting, and the resultant fluid from both was subjected to RNA isolation by the RNAsnap method (18). RNA was sequenced with 100-bp paired-end read (Illumina Hiseq2000) at LGC’s Agowa Genomics, Berlin, Germany. The library for sequencing was prepared with a NEB Ultra II RNA custom kit (NEB). Sequencing quality was assessed on the basis of average base quality, GC content, and adapter contamination as described previously (19). RNA sequences of adhered and unattached bacterial strain 4972 are available in the NCBI BioProject database under accession number PRJNA868552. Both alignment and feature counting were performed using the R package Rsubread (20). Differentially expressed genes (DEGs) were identified with a 0.05 false discovery rate (FDR) threshold using the exact test of edgeR (21, 22). Leading logFC values were used to generate an MDS plot using ggplot2 (23).

Sequences of DEGs unannotated by PROKKA (*n* = 148) along with annotated target genes were analyzed by NCBI blastp, Protein Homology/analogy Recognition Engine (Phyre2) (24), bacterial localization prediction tool (PSORTb 3.0) (25), transmembrane topology and signal peptide predictor (Phobius) (26), and Prediction of transmembrane helices in proteins (TMHMM Server v.2.0.) (27). DEGs were functionally annotated using eggnog-mapper (28) and assigned functions/categories according to the database of Clusters of Orthologous Genes (COGs) (29, 30). Next, the gene set enrichment analysis (GSEA) function from the clusterProfiler package was used to perform a gene set enrichment analysis for COG functions/categories (31). COG functions/categories were considered significantly upregulated/downregulated if Benjamini-Hochberg corrected *P* value was equal to or lower than 0.05.

### Bacterial gene deletion mutant generation

The Datsenko-Wanner recombineering method was applied with minor modifications for gene deletion of *flgH* and *spy* genes (32). For the deletion of *fimH*, *ffp*, *yggT*, *pilV,* and *spnT*, a method by Derous and Deboeck (33) was utilized. Positive clones were streaked on agar plates with appropriate antibiotics, and colony PCRs were performed to check and confirm successful gene disruption. The primers used are all listed in Table S4.

The whole genome of the gene deletion mutants was extracted by Qiagen Cube using the Qiagen DNA extraction kit and was sent to Novogene, UK for whole-genome sequencing with Illumina NovaSeq 6000 (Illumina). For single nucleotide variation search, the reads obtained for each isolate were mapped against the strain 4972 genome using bwa and called with bcftools (34). In the case of Δ*spnT*, one mutation before the start codon of the gene *nemR* was detected and was used for further analysis as it did not exhibit any irregularities in the growth of the strain. Sequences of all the deletion mutants for this study are available in the NCBI BioProject database under accession number PRJNA880721. The antibiotic resistance cassette in deletion mutants was removed using the pCP20 plasmid as described previously (35).

### Cloning of genes from strain 4972 into pBAD33 plasmid and complementation in mutants

For complementation of deletion mutants, only mutants exhibiting altered adhesion in initial experiments, i.e., *flgH*, *ffp, yggT*, *spnT,* and *pilV,* were cloned from the wild-type strain 4972 into expression vector pBAD33 as described previously (36). Five mutants (i.e., Δ*flgH*, Δ*ffp,* Δ*yggT*, Δ*spnT,* and Δ*pilV*) were transformed with a plasmid carrying corresponding complementing gene as well as pBAD33 vector plasmid alone (without insert) as a negative control (35).

### Minimum inhibitory concentration assay

Minimum inhibitory concentration (MIC) assay of the wild-type strain 4972 and gene mutants, i.e., Δ*ffp,* Δ*yggT,* Δ*spnT,* and Δ*pilV,* was carried out on a range of antibiotic concentrations of ampicillin (0–32 μg/mL) and gentamicin (0–8 μg/mL). For this, 1 mL of bacterial strain culture was incubated for 16 h at 37°C and 180 rpm. The OD_600_ was determined, and bacteria were diluted in the infection media to get a total volume of 1 mL containing 5 × 10^6^ bacteria. This was then aliquoted to 200 µL per well into a 96-well Nunclon plate and incubated at 37°C. After the 24 h, OD_600_ was measured for each well with the Tecan microplate reader (Tecan, Austria). Three technical and biological repetitions were performed for each strain. The lowest concentration inhibiting growth, i.e., MIC, was defined as OD_600_, where no growth was observed, mirroring the optical density of the negative control.

### Motility assays

Motility assays of the wild-type strain 4972, deletion mutants, complemented mutants, and mutants with empty plasmid were conducted according to the already published protocol (17, 36).

### Prevalence of target genes in different pathotypes

Genes targeted for deletion were analyzed for prevalence in various *E. coli* pathotypes using Abricate and *E. coli* genomes downloaded from the RefSeq database (accessed on 26 October 2021) (37). The 25,436 downloaded genomes were filtered to exclude those of low quality (coverage below 50, third generation, or unknown sequencing platform, more than 400 contigs) and then pathotyped by running a BLAST search of selected genes associated with various pathotypes against the database of genomes. Genes with *E*-value ≤ 10^−100^ were considered as present (13). Decisions used for pathotyping and the numbers of genomes assigned to each pathotype are shown in Table S5.

### Global proteome analysis of *E. coli* clumps

For the proteomic analysis of clumps and planktonic bacteria, the adhesion assay of the wild-type strain 4972 on cell line 5637 was carried out as described above. The planktonic bacteria in the supernatant were removed from each well of the 24-well plate and pooled using a pipette. The adhered bacterial cells were carefully detached by aspirating ice-cold 1× PBS in each well of the 24-well plate using a pipette. Bacterial cells were washed with ice-cold 1× PBS and finally resuspended in 1 mL of 1× PBS. Bacterial cells were resuspended in freshly prepared 8 M urea lysis buffer (8 M Urea, 5 mM EDTA, 100 mM NH_4_HCO_3_, and 1 mM DTT, pH 8.0) and lysed by sterile bead (500 mg glass beads) beating for 3 min with a frequency of 30 s^−1^ by TissueLyser II (Qiagen). The experiment and protein extraction were carried out five times independently, i.e., five biological replicates.

Protein extracts were sent to the Bavarian Center for Biomolecular Mass Spectrometry (BayBioMS) for proteomic analysis. Per sample, 15 µg of protein extract was reduced (10 mM DTT), carbamidomethylated (55 mM CAA), and digested with trypsin (0.3 µg Trypsin Gold, Promega). Generated peptides (0.2 µg per injection) were analyzed on a Dionex Ultimate 3000 RSLCnano system coupled to a Q-Exactive HF-X mass spectrometer (ThermoFisher Scientific) using a 50 min linear gradient from 4% to 32% of solvent B [0.1% (vol/vol) formic acid and 5% (vol/vol) dimethyl sulfoxide (DMSO) in acetonitrile] at 300 nL/min flow rate. nanoLC solvent A was 0.1% (vol/vol) formic acid and 5% (vol/vol) DMSO in HPLC-grade water. The Q-Exactive HF-X mass spectrometer was operated in data-dependent acquisition and positive ionization modes. Peptide fragmentation was performed using higher energy collision-induced dissociation and a normalized collision energy of 26%.

Peptide identification and quantification were done using the software MaxQuant (version 1.6.3.4) (38). MS2 spectra were searched against the proteins from PROKKA annotated strain 4972 whole genome, supplemented with common contaminants (built-in option in MaxQuant). The results were adjusted to 1% FDR on peptide spectrum match level and protein level, employing a target-decoy approach using reversed protein sequences. The proteins differentially expressed between adhered and planktonic bacterial cells were identified using the label-free quantification algorithm provided by MaxQuant (LFQ) (39) and further analyzed with the limma R package. Briefly, the data provided were processed by first filtering out the peptides, which equaled 1. Rows with missing intensity values for at least four samples were filtered, and LFQ intensity values were logarithmized (log2). The missing values were imputed using the MinDet method from imputeLCMD. A linear model was fitted to the data using moderated *t*-statistics, and log odds of differential expression were calculated by empirical Bayes moderation of the standard errors toward a global value using the limma package. *P*-values were adjusted for multiple testing using the Benjamini-Hochberg method. DEGs were identified with a 0.05 false discovery rate threshold and logFC values below −0.5 or above 0.5. The proteins were functionally annotated with the use of an eggNOG-mapper and used for gene set enrichment analysis, as mentioned previously in section “Biofilm assay” (28).

### Statistical analyses

Statistical analysis was carried out using the R statistical software (40). Gene prevalence was compared using the Chi-squared test of independence implemented in R. Figures were generated with the use of the ggplot2 package implemented in R software (41). Wilcoxon rank-sum test was used to analyze the differences, and Benjamini-Hochberg (*P*.BH) lower than 0.05 was considered significant.

## RESULTS

### Clump formation by *E. coli* 4972 is specific to epithelial cells

**Fig 1:**
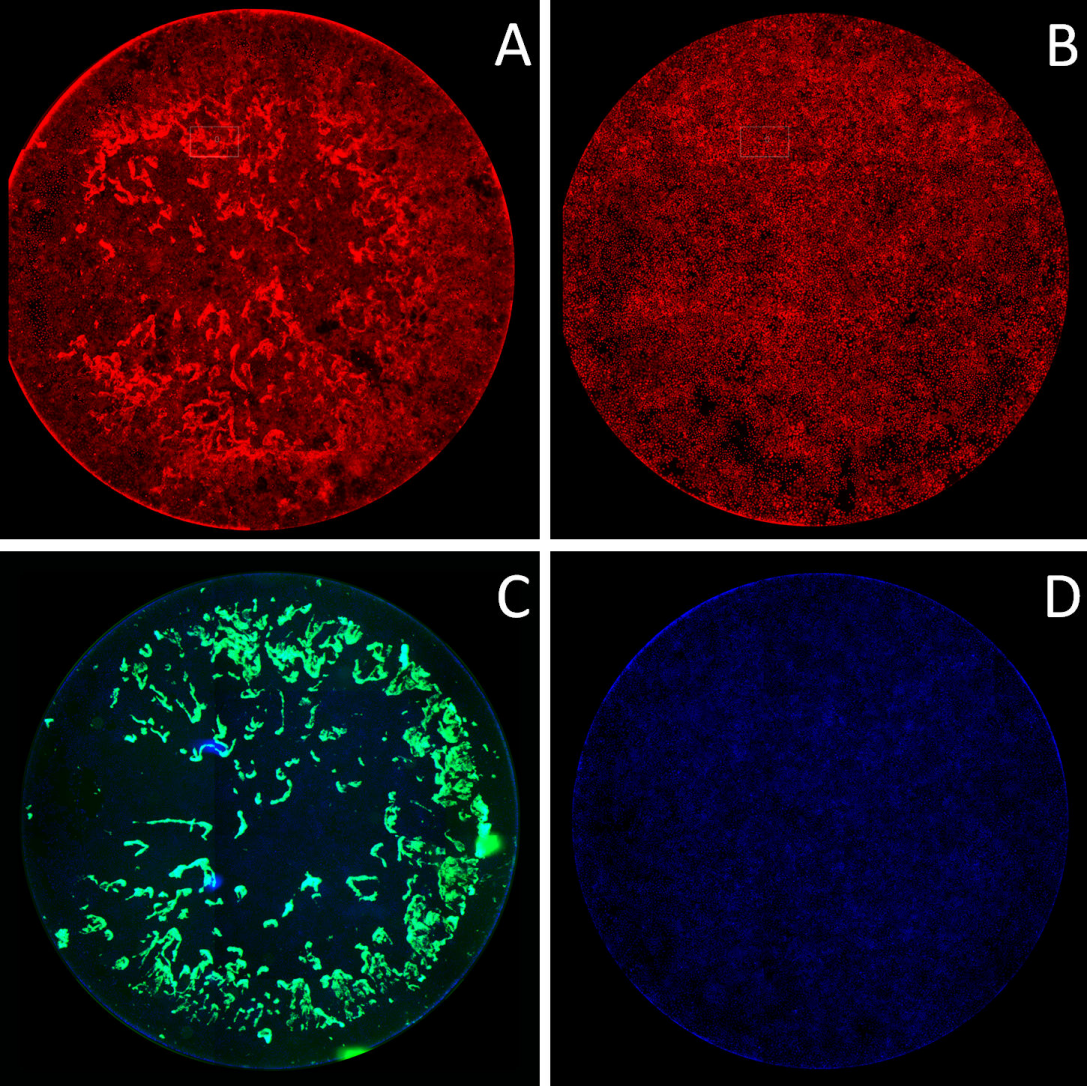
Clump formation by strain 4972. (**A**) PI-stained wild-type *E. coli* strain 4972, (**B**) PI-stained 5637 cell line only as a negative control, (**C**) GFP-expressing 4972 + pFPV25.1 Kan and DAPI-stained nuclei of 5637 cell line, and (**D**) DAPI-stained nuclei of 5637 cell line only as a negative control, after 4-h incubation of adhesion assay and visualized by Aklides system.

We previously defined a new adhesion phenotype that was demonstrated by two *E. coli* strains, one isolated from human, i.e., 4972, was used in this study, and the other from squirrel. The adhesion pattern exhibited a biofilm-like structure, referred to as clumps, within 4 h on human urinary bladder epithelial cell line 5637 (10). Clumps were less pronounced on PK15 and Caco-2 cell lines. *E. coli* strain 4972 formed a weak biofilm on plastic wells (Fig. S1) but an extensive clumpy structure on the 5637 bladder cell line (Fig. 1).

### Strain 4972 shares phylogenomic placement with diverse *E. coli* pathotypes

**Fig 2:**
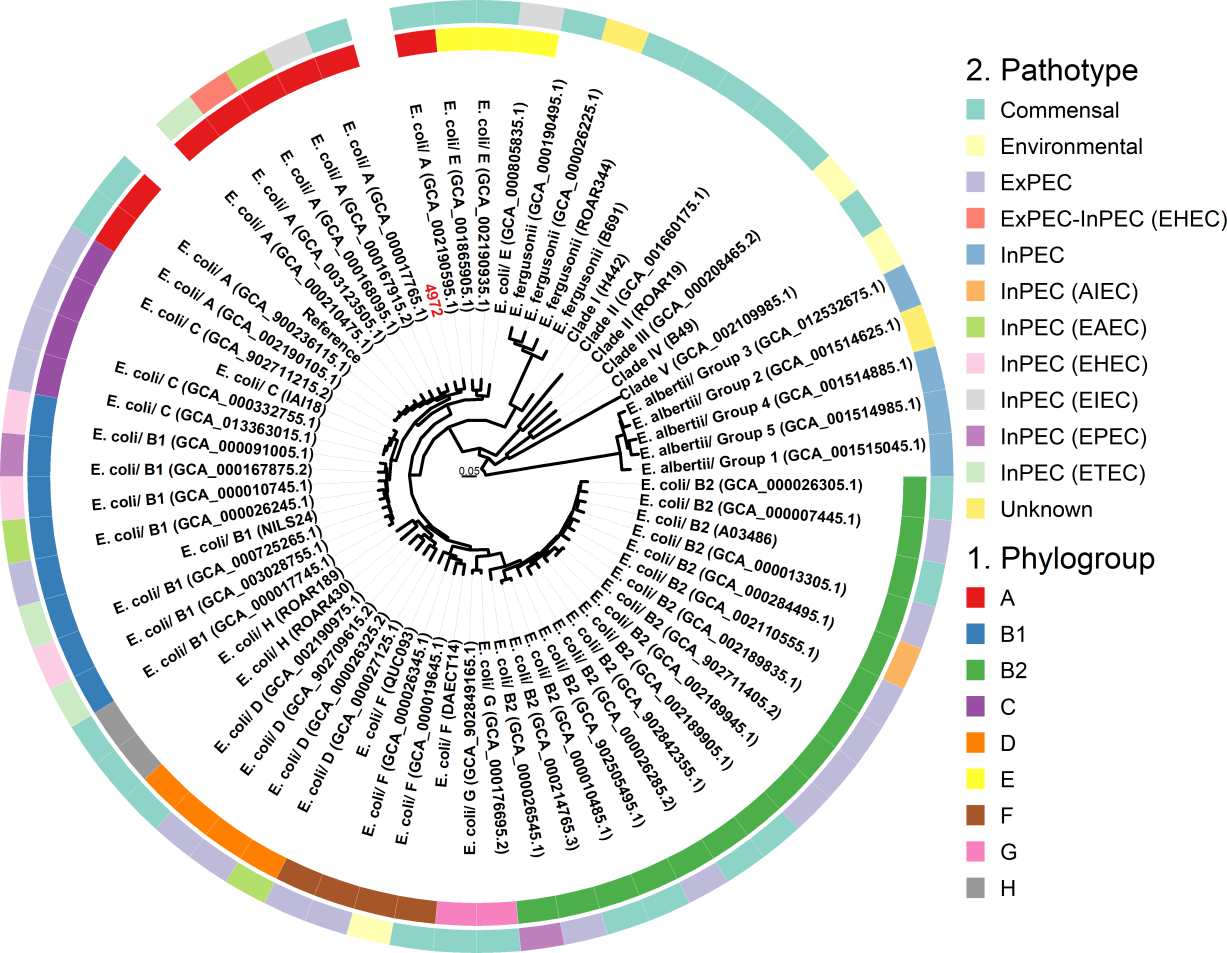
Genomic comparison of *E. coli* strain 4972 phylogroup and pathotypes compared to already published 66 reference genomes of pathogenic and commensal *E. coli* and annotated on the tree with the use of ggtree package. *E. coli* K-12 MG1655 was used as a reference for mapping and snp calling. Placement of the strain 4972 on the tree is highlighted in red.

The phylogenetic relationship of *E. coli* strain 4972 was investigated in the context of a diverse collection of *E. coli* reference strains from multiple phylogroups and pathotypes, including commensal strains. A phylogenetic tree was constructed based on SNPs in the shared genome with reference to *E. coli* K-12 MG1655 (Fig. 2). *E. coli* strain 4972 was present within a clade corresponding to phylogroup A (14) containing strains isolated from human fecal samples with diverse lifestyles, including commensal, enteroinvasive *E. coli* (EIEC), EAEC, enterohemorrhagic *E. coli* (EHEC), and enterotoxigenic *E. coli* (ETEC) pathotypes.

### Transcriptome reveals differences among the subpopulation of sessile bacteria constituting the clumps and planktonic bacteria in the supernatant

**Fig 3:**
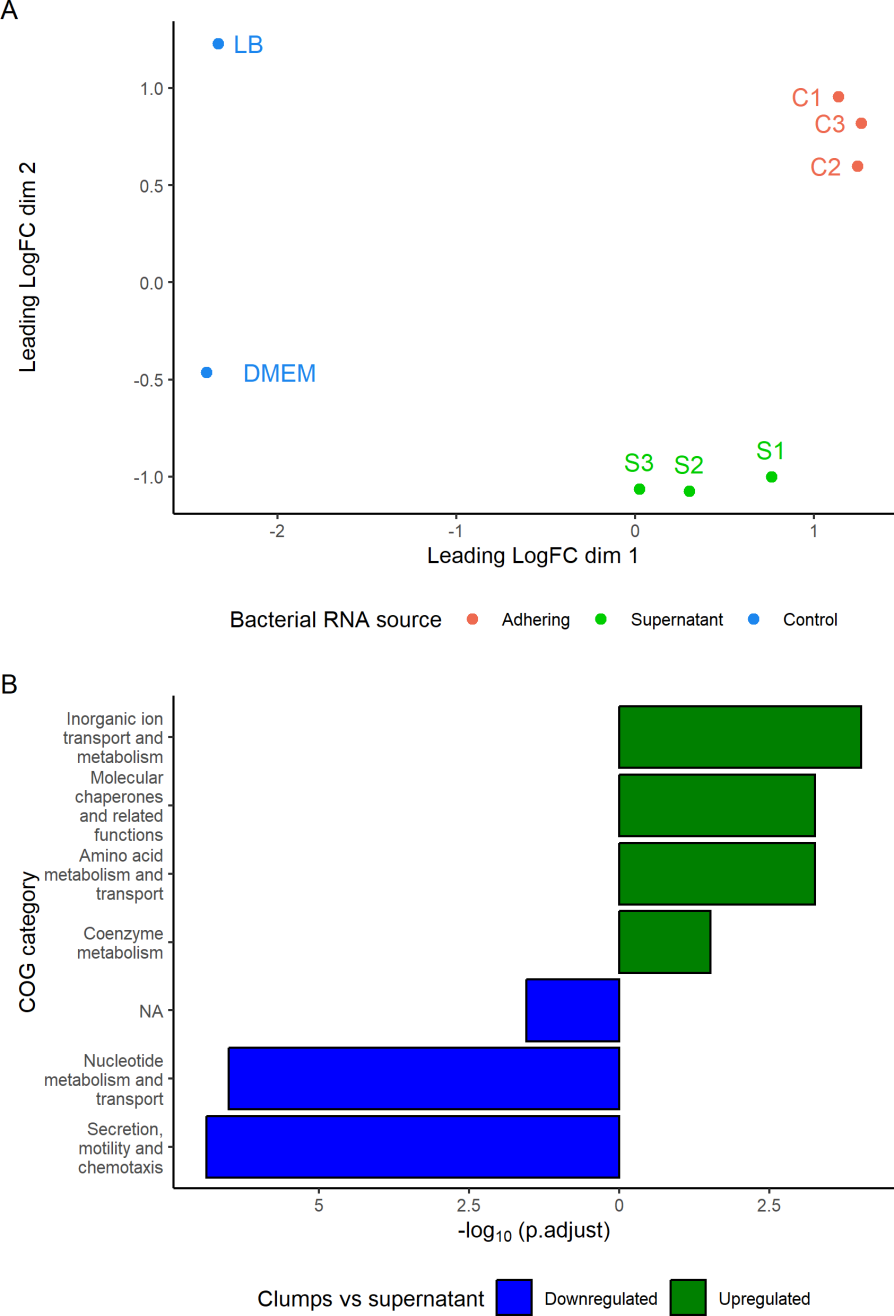
Differential gene expression of adhered and un-adhered bacteria. (**A**) Multidimensional scaling plot of distances between the samples of RNA sequencing, and leading log-fold change is the root mean square average of the largest log2-fold changes between each pair of samples. The clumps on the cell lines 5637, PK15, and CaCo-2 are referred to as C1, C2, and C3, respectively. Planktonic bacteria in the supernatant on the cell lines 5637, PK15, and CaCo-2 are labeled as S1, S2, and S3. (**B**) Differentially expressed significant COGs were compared between adhered and planktonic clumpy *E. coli* for all genes that had *P*.BH value lower than 0.05 in edgeR analysis. Absolute logarithm of Benjamini-Hochberg-adjusted *P* values from GSEA is on the *x*-axis, and COG categories are shown on the *y*-axis. NA category corresponds to genes that were not assigned to any of the COGs.

To identify potential mechanisms of clump formation, we first investigated genes that were differentially expressed in bacterial cells present in clumps associated with host cells and compared them with planktonic cells in the culture medium that were not associated with host cells. Overall RNA expression profiles of bacteria on 5637, PK15, and Caco-2 cell lines clustered based on the subpopulations of adherent bacteria (C1–C3) or planktonic bacteria (S1–S3) and not the host cell line (Fig. 3A). This was consistent with the idea that during bacterial attachment to the host cell, strain 4972 may utilize similar processes of adhesion on different cells. Therefore, RNAseq data of the *E. coli* strain 4972 on the three different cell lines were pooled, and data analysis revealed a total of 622 differentially expressed genes between adherent and planktonic bacteria.

To investigate whether distinct functional classes of DEGs were differentially expressed in clumps compared to planktonic culture, DEGs were assigned to functional categories based on sequence similarity (Fig. 3B). Four functional classes contained significantly more genes that were expressed at a higher level and three classes at a lower level in clumps compared to planktonic cells. “Molecular chaperones and related functions” class, “Inorganic ion transport and metabolism” class, “Amino acid metabolism and transport” class, and “Coenzyme metabolism” class were enriched in clumps (*P*.BH < 0.05). In contrast, flagellar and fimbriae genes represented by “Secretion, motility, and chemotaxis” COG class and “Nucleotide metabolism and transport” contained significantly more genes that were enriched in bacteria in the supernatants (*P*.BH < 0.05), whereas curli genes (*csgABCDEFG*) remained undifferentiated in the two states. Furthermore, genes that did not fulfill the criteria of any COG category (“NA,” Fig. 3B) were represented by significantly more genes expressed at a lower level in clumps (*P*.BH < 0.05).

Seven genes that exhibited differential expression in clumps compared with planktonic, *ffp, pilV, spnT, yggT, spy, flgH,* and *fimH,* were chosen for further analysis. The selection was carried on the basis of transcriptomic differential expression, i.e., three genes that were expressed at a higher level and four at a lower level in clumps. Additionally, diverse putative functions and predicted subcellular localization were also taken into account along with other sequence-based analyses (Table 1). In terms of prevalence, the seven targeted genes had variable distribution within pathotypes and commensal *E. coli* (Fig. S2). The *flgH, spy,* and *yggT* genes were widely distributed, present in at least 95% of strains in each pathotype. The *ffp* and *fimH* genes were also widely distributed, present in more than 80% of strains in each class, with the exception of DAEC, typical EPEC (tEPEC), and uropathogenic *E. coli* (UPEC) for *ffp*, and EAEC pathotype for *fimH*. The *pilV* and *spnT* genes were rare in all pathotypes of *E. coli*. The *spnT* gene was present in less than 5% of six pathotypes and commensal strains and absent from EHEC, EIEC, and STEC. The *pilV* gene was present in 17 out of 5,259 (0.32 %) nonpathogenic *E. coli* strains and 1 out of 2,598 (0.038 %) UPEC strains.

**TABLE 1:** Sequence-based analysis of target genes from transcriptomic data

Gene	Log FC	Putative function	Predicted location	Phobius signal peptide^*a*^	Phyre2 molecule	TMHMM helices^*a*^	COG^*b*^ category
*ffp*	1.59	Fimbrial family protein	Cytoplasm	−	Type-1 fimbrial protein	−	N
*pilV*	−1.12	Conjugative pilus tip adhesin	Extracellular	n11-22c27/28o	Fimbrial protein	−	N
*spnT*	−0.88	***spnT***	Cytoplasm	−	Chaperone of ribosomal protein I4	−	NA
*yggT*	1.36	Osmotic shock tolerance protein	Cytoplasmic membrane	−	Gamma-aminobutyric acid type b receptor	Yes	S
*spy*	6.57	***spy***	Periplasmic	n4-15c23/24o	Periplasmic protein related to spheroblast formation	−	N, P, T
*flgH*	−2.47	Flagellar basal body L-ring protein	Outer membrane	n14-25c33/34o	Flagellar l-ring protein	−	N
*fimH*	−2.44	Type 1 fimbrial adhesion	Extracellular and cytoplasmic membrane	n4-14c19/20o	Fimh protein	−	N, U

^*a*^ −, not detected.

^*b*^ N, secretion, motility, and chemotaxis; O, molecular chaperones and related functions; P, inorganic ion transport and metabolism; T, signal transduction; U, intracellular trafficking, secretion, and vesicular transport; S, unknown function; and NA, unassigned COG category.

### Deletion of *flgH* and complementation of *yggT* significantly diminishes adhesion and clump formation on 5637 cells

**Fig 4:**
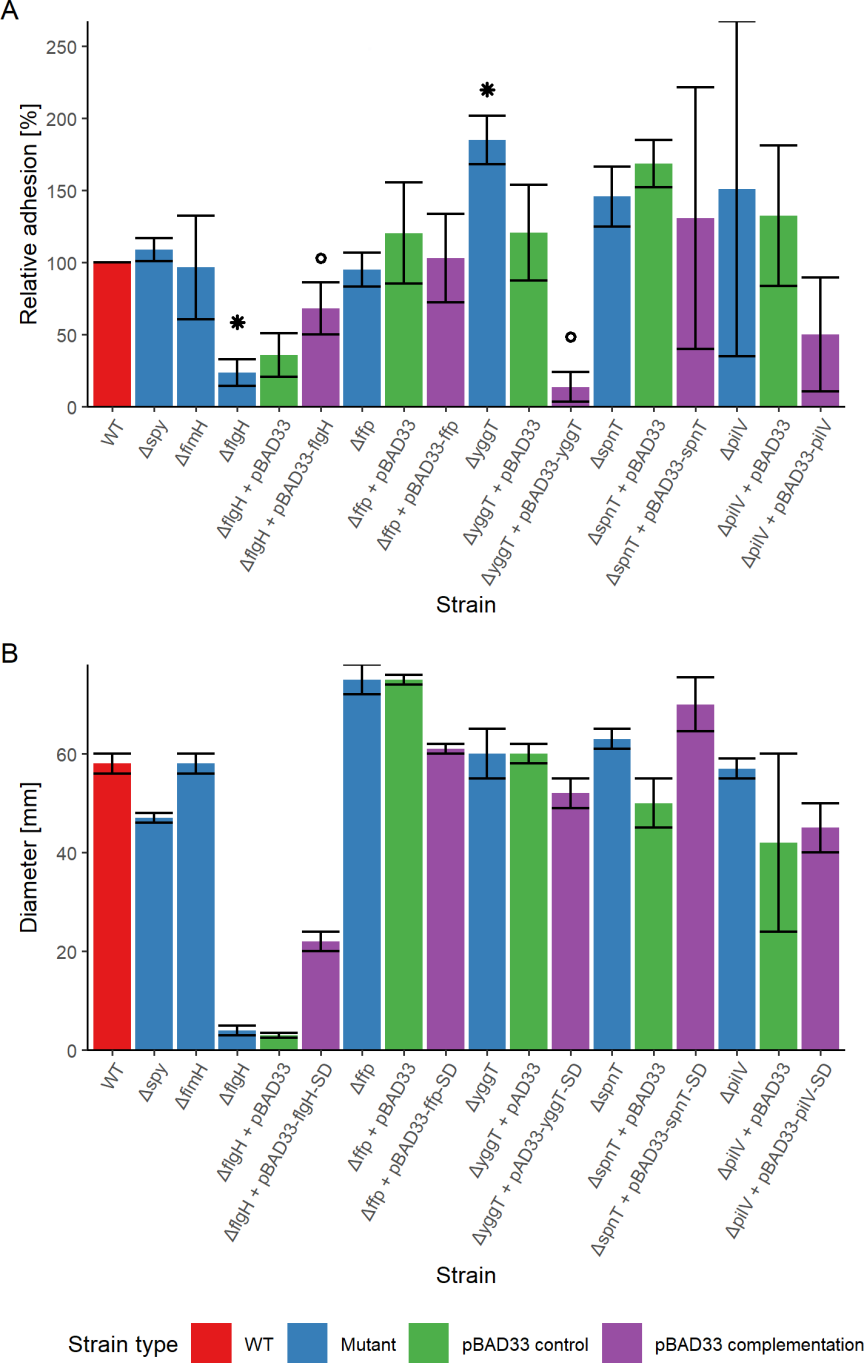
(**A**) Adhesion and (**B**) motility assays. (**A**) Adhesion to 5637 bladder cell line of WT (median value normalized to 100%), gene deletion mutants (Mutant), complemented mutants (pBAD33 complementation), and deletion mutants with empty plasmid (pBAD33 control). The data are shown as median values and median absolute deviation (MAD) of five separate experiments of adhesion assay. *X*-axis shows the strains, and *y*-axis depicts relative adhesion (%). Wilcoxon rank-sum test was used to determine the significance between different strains. Statistically significant results indicating changes in mutant adhesion compared to the WT are denoted by an asterisk (*), and significant changes observed upon complementation of the mutant are marked with a small circle (○). (**B**) Motility of WT (median value normalized to 100%), gene deletion mutants (Mutant), complemented mutants (pBAD33 complementation), and deletion mutants with empty plasmid (pBAD33 control). The data are shown as median values and MAD of three different experiments with three replicates. *X*-axis shows the strains, and *y*-axis depicts the diameter of the zone of bacteria. Wilcoxon rank-sum test was used to determine the significance between different strains.

To investigate the role of various genes on the number of bacteria comprising the clumps when adhered to the 5637 cell line, we constructed isogenic strains in which each gene was individually replaced by a resistance gene. Only Δ*flgH* exhibited a significant (*P*.BH < 0.05) reduction in adhesion, whereby the mutant’s ability to adhere was decreased by up to 80% when compared to the wild type. This mutant was also the only one with visible change, showing reduction in clump formation on the 5637 cell line (Fig. S3). The reintroduction of the *flgH* gene in Δ*flgH,* i.e., Δ*flgH* + pBAD33 *flgH* resulted in significant (*P* = 0.05) regeneration of clumpy adhesion up to 70%, confirming the role of flagella in adhesion. Δ*ffp* and its complementation, i.e., Δ*ffp* + pBAD33 *ffp* had no significant impact on adhesion. Δ*spy* and Δ*fimH* had no significant change in the adhesion ability on the 5637 bladder cell line. Deletion of *yggT* had a statistically significant (*P* < 0.05) increase in adhesion up to 184% that of the wild-type strain (Fig. 4A). Δ*yggT* and its respective introduction via pBAD33 system significantly (*P* < 0.05) decreased adhesion to 13%. In the case of Δ*pilV,* complementation in *trans* did result in a decrease to 53% adhesion, but it was not statistically significant. Similarly, the deletion of *spnT* resulted in an increase to 146% adhesion, but neither the deletion nor complementation of this gene caused a statistically significant change in adhesion to the 5637 cells.

### Deletion of *flgH* rendered the strain non-motile, and *ffp* deletion increased motility

Motility is implicated in both biofilm formation and adhesion (36) and to further investigate the role of genes affecting adhesion and the clump phenotype, motility in soft agar was determined (Fig. 4B). Only Δ*flgH* lost motility and increased significantly (*P*.BH < 0.05) when complemented in *trans*. Δ*ffp* had significantly (*P*.BH < 0.05) increased motility and when we compared Δ*ffp* + pBAD33 with the complemented strain, i.e., Δ*ffp* + pBAD33 *ffp,* motility was reduced to the wild-type level (*P*.BH < 0.05). Deletion of *pilV, spnT, yggT,* and *fimH* did not significantly affect motility and remained unchanged throughout the assays (Fig. 4B). The deletion of the *spy* gene caused a significant reduction in motility (*P*.BH < 0.05) when compared to the wild-type strain, although the mutant remained motile.

### *ffp* and *spnT* mutants are highly tolerant to ampicillin, and *yggT* mutant is highly susceptible to gentamicin

As the clump formation exhibited similarity to a biofilm-like 3D structure, we addressed the hypothesis that the clump structure protected the bacteria against external stresses. We tested this hypothesis using an adhesion assay in the presence of antibiotic stress. The study not only examined the antibiotic action on the wild-type strain forming the clumpy structure but also elucidated the difference in the survival of viable bacteria of the biofilm-like structure formed by the four gene-deletion mutants, i.e., Δ*ffp,* Δ*yggT*, Δ*spnT,* and Δ*pilV,* whose deletion resulted in a general increase in the adhesion percentage. The antibiotics with different modes of action against *E. coli* were selected to stress the clumpy structure. The clumps formed by wild-type strain 4972 were exposed to two different antibiotics, ampicillin and gentamicin, and the concentrations determined were 16,000 and 2 µg/mL, respectively, as depicted by the results of antibiotic dose-ranging experiments (Fig. S4). The exposure to these antibiotic concentrations resulted in a reduction of more than 50% of the viable bacteria in the clumps of the wild-type strain.

**Fig 5:**
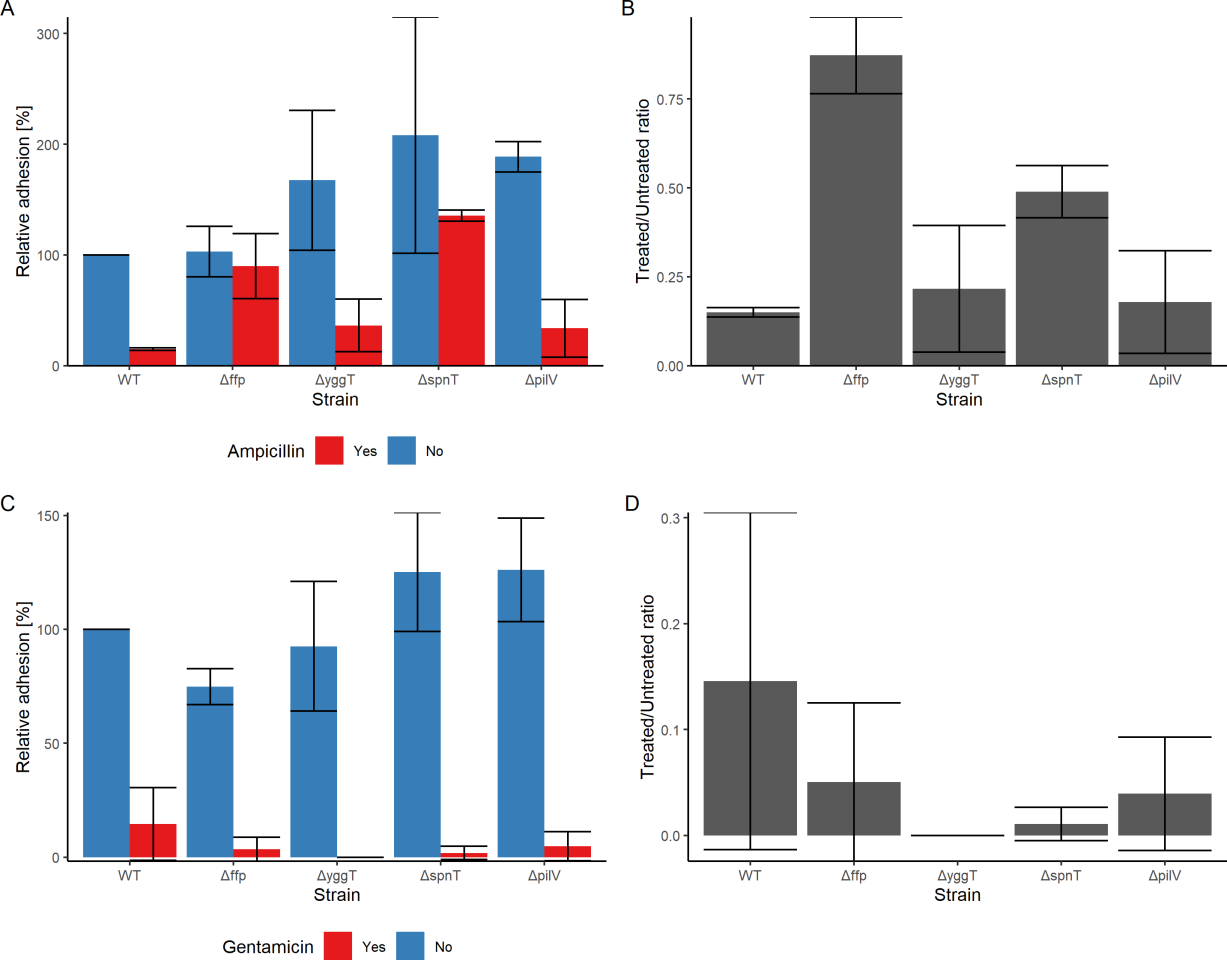
Survival of bacteria in clumps after antibiotic stress. (**A**) WT without ampicillin stress (median value normalized to 100%), and the four genes are shown with (red bar) and without (blue bar) ampicillin when adhered to the 5637 cell line. (**B**) Ampicillin-treated/untreated bacteria ratio for WT along with four genes when adhered to the 5637 cell line. (**C**) WT without gentamicin stress (median value normalized to 100%), and the four genes are shown with (red bar) and without (blue bar) gentamicin when adhered to 5637 cell line. (**D**) Gentamicin-treated/untreated bacteria ratio for WT along with four genes when adhered to 5637 cell line. The stress concentrations used for ampicillin and gentamicin are 16,000 and 2 µg/mL, respectively. The data comprise median values and median absolute deviation of five separate experiments in triplicate (**A and B**) or ratio of median values for antibiotic-treated and untreated bacteria (**C and D**). The strain names are on the *x*-axis, and the relative median adhesion is shown on the *y*-axis. Wilcoxon rank-sum test with *P*.BH < 0.05 was used to determine the significance between different strains.

In case of ampicillin exposure, clumps produced by Δ*ffp* and Δ*spnT* were significantly (*P*.BH < 0.05) more resistant and protected the bacterial clumps, with Δ*spnT* showing the highest number of viable bacteria in terms of relative adhesion as exhibited in Fig. 5A. It is also surprising to note that the viable bacteria after ampicillin action on the clumpy structures by Δ*ffp* and Δ*spnT* did not significantly differ from the wild-type strain. It is also important to note that Δ*spnT* maintained the best overall bacterial density in media with and without ampicillin, whereas Δ*ffp* provided the greatest relative protection from ampicillin (Fig. 5B). In the case of gentamicin stress, clumps built by Δ*yggT* were highly susceptible, and no viable bacteria were detected after gentamicin exposure. Both Δ*spnT* and Δ*yggT* had significantly (*P*.BH <0.05) low percentage of survival under gentamicin stress when compared to the wild type. In contrast, Δ*ffp* and Δ*pilV* production of clumpy structure variedly protected the bacteria against the stress, similar to the wild-type strain; therefore, the decrease is not significant (Fig. 5C and D).

Wild-type strain 4972, Δ*ffp,* Δ*yggT*, Δ*spnT,* and Δ*pilV* were also tested for sensitivity against the antibiotic stress in the planktonic state, i.e., in the absence of host eukaryotic cells in MIC assays. Antibiotics inhibited growth at a very low concentration of ampicillin (8 µg/mL) and gentamicin (0.5 µg/mL). Furthermore, the growth declined with an increase in ampicillin concentrations and did not differ between wild type and mutants as shown in Fig. S5A. Stress with gentamicin depicted similar results, where it significantly inhibited the growth as shown in Fig. S5B. Only Δ*yggT* was significantly (*P* < 0.05) more susceptible at the gentamicin concentration of 0.25 µg/mL.

### Global proteome analysis

In order to add another omics layer, a global proteomics analysis of the wild-type strain was carried out for adhered and planktonic bacteria. The aim was to get a clearer picture of the differential expression of proteins involved in sessile and planktonic populations. As a result of this comparison, 325 proteins were expressed at a higher level in bacteria in the supernatant as compared to 422 proteins having higher expression in the adhering state of bacteria, as shown in the volcano plot (Fig. S6). It was also interesting to note that three genes (i.e., *flgH*, *spy,* and *spnT*) out of seven genes targeted in this study were detected at the proteome level, and two of them (*spy* and *spnT*) were significantly differentially expressed (*P*.BH < 0.05).

**Fig 6:**
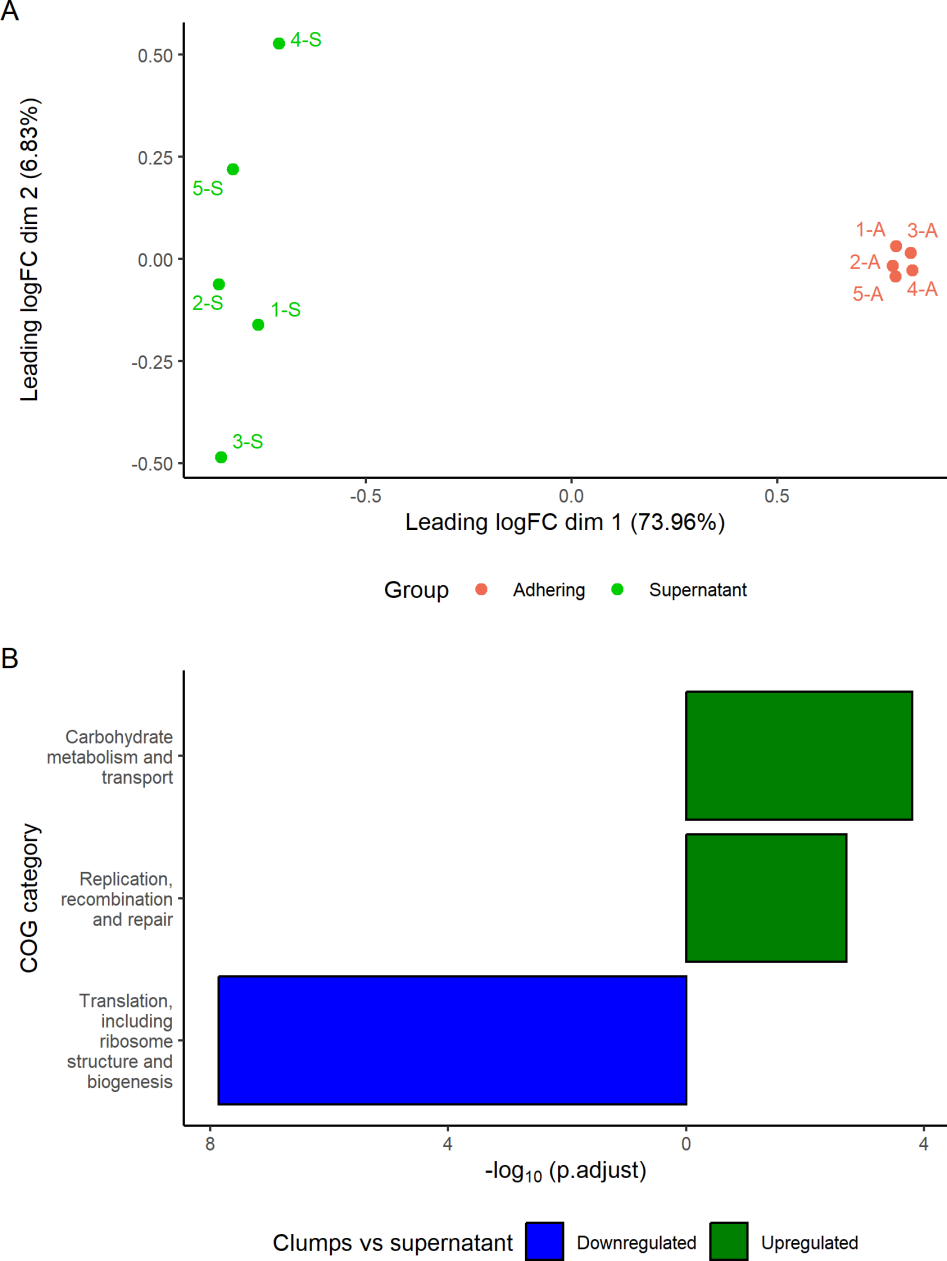
Differential protein expression of adhered clumps and bacteria in the supernatant. (**A**) Multidimensional scaling plot of distances between the samples, and leading log-fold change is the root mean square average of the largest log2-fold changes between each pair of samples. (**B**) Significantly differentially expressed proteins categorized on the genes/proteins in the genome. COGs were compared between adhered and planktonic clumpy *E. coli* for all genes that had *P*.BH value lower than 0.05 and logFC values below −0.5 or above 0.5 in limma analysis. Absolute logarithm of Benjamini-Hochberg adjusted *P* values from GSEA is on the *x*-axis, and COG categories are shown on the *y*-axis.

COGs were compared between clumps and planktonic bacteria (Fig. 6). The groups of proteins categorized as “Translation, including ribosome structure and biogenesis” were significantly (*P*.BH < 0.05) downregulated group of COG in the adherent bacteria. The proteins categorized as “Carbohydrate metabolism and transport” and “Replication recombination and repair” were significantly enriched (*P*.BH < 0.05). Additionally, specific flagellar proteins such as *flgE* and *fliG* were downregulated in the clumps.

## DISCUSSION

The study characterized the biofilm-like 3D structure of clumps formed during adhesion by *E. coli* strain 4972 to different epithelial cells. Normally, biofilm formation is studied on synthetic or plastic materials as it is difficult to quantify them on the living cells or structures. The strain was tested in multiple media to rule out media or synthetic/plastic surfaces, influencing clump formation as the strain strictly formed these structures on the epithelial cells and most prominently on the human urinary bladder cells. We have taken advantage of the unique ability of this strain and characterized it using omics technologies. Clump formation on the cell surface and the effects on the antimicrobial resistance were also studied. Clumps exhibited the characteristics of a biofilm where the adhered or sessile bacterial population on the cell line was shielded from stress by two different antibiotics in contrast to unattached or planktonic bacteria. Hence, the adhesion phenotype, similar to biofilm served as a physical barrier, and the thickness and composition allowed the bacteria to bear a high concentration of antibiotics (42) in contrast to bacteria in the planktonic state, which leaves them vulnerable to antibiotic action. Inherently, the clumpy *E. coli* is sensitive to both antibiotics as shown by the MIC assay. It is important to note that the parameters of the MIC assay and adhesion assay with antibiotic stress are widely different and cannot be compared directly, but it is necessary to highlight the peculiar behavior of the clumpy adhesion phenotype, which allows the bacteria to survive an extremely high concentration of one antibiotic, i.e., ampicillin (16 mg/mL), while leaving them more susceptible to another, i.e., gentamicin (2 µg/mL) at a lower concentration.

RNAseq data and global proteome analysis comparing unattached and adhered bacteria showed DEGs and differentially abundant proteins in each state. Transcriptomes and proteomes of bacteria in clumps clustered together and diverged from transcriptomes and proteomes of bacteria in the supernatant. Transcriptomes of both pathogenic and nonpathogenic *E. coli* with the ability to form biofilm have been studied earlier (*E. coli* K-12, symptomatic bacteriuria *E. coli,* and ocular *E. coli*) using various platforms. It was reported that overall motility and fimbriae genes are downregulated in the transcriptome when *E. coli* adhere to host cells and form biofilms (43, 44). Both flagella genes (i.e., *flgBCDEFGHIJ*) and fimbriae genes (i.e., *fimACDEFGHI*) were downregulated in the clumps, which is in line with the gene expression of symptomatic bacteriuria *E. coli* strains 83972 and VR50 during biofilm. In both cases, clump formation and biofilm, none of the curli genes, *csgABCDEFG*, were differentially expressed at the transcriptomic level (45). Genes categorized as “Inorganic transport and metabolisms,” and “Coenzyme transport and metabolism,” were significantly upregulated in clumps, and similar expression pattern was observed in biofilms by *E. coli* K-12 and symptomatic bacteriuria *E. coli*. Post-translational modification, protein turnover, and chaperons were upregulated in clumps, but the expression varied in *E. coli* K-12 but was the opposite in the cases of symptomatic bacteriuria *E. coli* and ocular *E. coli* (45–48).

The study also elucidates the contribution of factors crucial to clump formation to the susceptibility of the selected antibiotics. Based on RNAseq data, gene candidates (i.e., *flgH*, *fimH*, *spy*, *ffp*, *yggT*, *spnT*, and *pilV*) were selected for gene deletion and characterized. The flagella genes were downregulated at both transcriptomic and proteomic levels. The target gene *flgH* encodes flagellar L-ring protein, which is the basic subunit making up the lipopolysaccharide ring of the flagellar basal body and lies in the outer membrane and encircles the basal body rod of the flagella. L ring is embedded in the cell wall, and the flagellar hook (encoded by *flgE*) does not proceed until the P ring (encoded by *flgI*) and L ring are assembled, rendering the bacteria nonmotile (49). Upon adhesion to the 5637 cell line, Δ*flgH* did not develop a clumpy phenotype and had lost the ability to adhere. This is in accordance with other studies where functional flagella were found as the basis for adhesion to host cells. It has also been reported that a strong relationship exists in *E. coli* between motility, flagella, and biofilm architecture (50, 51). Type 1 fimbriae were downregulated at the transcriptomic level and showed the same behavior at the proteome level. However, Δ*fimH* exhibited no effect on adhesion and motility. These results may be a consequence of the compensatory effect, in which suppressed expression of one fimbrial cluster may result in the overexpression of other adhesins and consequently maintain the adhesion capacity of the deletion mutant to a cell line or tissue evading change detection (6). Similar to our results, several *E. coli* FimH mutants remained unaltered in adherence phenotype to epithelial cells when compared to parental strains (52, 53).

Protein folding genes were mostly upregulated, with *spy* periplasmic chaperone being significantly upregulated at both transcriptomic and proteomics levels. It encodes an ATP-independent periplasmic chaperone known for preventing protein aggregation and assisting protein refolding and is highly induced in biofilms (46, 54). The evaluation of the comparative assays found that the deletion of *spy* had no effect on clump formation, but it caused a reduction in motility. These results seem to be consistent with previously published research, as it has been shown that periplasmic chaperones seem to be almost functionally redundant; the loss of certain chaperones in deletion mutants could, therefore, be very well compensated, which may explain the lack of change in phenotype (55). In order to fully understand the role of chaperones, more mutants are required to be tested.

The fimbrial family protein or *ffp* gene upregulated in clumps was targeted for deletion. According to the Phyre2 tool, it is a structural protein with similarity to type 1 fimbrial proteins, but no functional information is available for this gene. Interestingly, Δ*ffp* was more motile compared to the wild type, and its overexpression significantly reduced motility. Similar characteristics are exhibited by the deletion of *papX*, another fimbrial gene (56). The overexpression of *papX* correlated with both a significant reduction in flagellin protein synthesis and flagella assembled on the cell surface. *papX* has been implicated in being the master regulator of flagellar synthesis and chemotaxis of UPEC CFT073 (56). When ampicillin stress was applied after the clump formation, Δ*ffp* resisted significantly better compared to the wild type, showing the greatest relative protection from ampicillin. The role of the *ffp* gene requires extensive research in the direction of being a regulator function relating to motility and leading to tolerance of ampicillin’s action of interference with cell wall synthesis or permeability in clumps or biofilm-like structures.

Pilus tip adhesin from the shufflon system or *pilV*, a downregulated gene, was identified in the RNAseq analysis. It has been reported that plasmid 1 of the Shiga-toxigenic hybrid strain 12-05829 carries a type IV pilus biosynthesis locus (*pil*) consisting of 11 genes (*pilL* to *pilV* and *pilI*) (57). Type IV pili are important virulence factors in many pathogenic Gram-negative bacteria, as they mediate adherence to eukaryotic cells and host colonization and are associated with other phenomena related to pathogenicity, including bacterial aggregation, biofilm formation, DNA binding and uptake, and twitching motility (58). Similar to our results of the adhesion assay, deletion of the *pil* locus did not affect bacterial auto-aggregation or adhesion to HEp-2 cells in strain 12-05829 and O113:H2 98NK2 strain (57).

Similar to Δ*ffp*, Δ*spnT* resisted ampicillin stress more than the wild-type strain under the same conditions. A concentration of 16 mg/mL of ampicillin did not exhibit a significant effect on bacteria in the clumps. In contrast, Δ*spnT* was highly susceptible to gentamicin with the lowest number of surviving bacteria in the clumps. Wei et al. (59) failed to disrupt *spnT* but studied its overexpression in *Serratia marcescens* and *E. coli* MG1655. The overexpression of *spnT* in *E. coli* MG1655 resulted in elongated cell morphology and nucleoids after arabinose-mediated induction of *spnT* expression. This indicated that enhanced expression of *spnT* increases the DNA content per *E. coli* cell and blocks the replication cycle in *E. coli,* possibly through the PriA pathway, causing bacterial DNA aggregation and inhibiting cell division. The study by Horng et al. (60) reported that overexpression of this gene in *S. marcescens* adversely affected pigment production and sliding motility. They were also unable to disrupt the gene by using homologous recombination so only reported its overexpression. In our case, it can be hypothesized that since overexpression inhibits cell division, deletion of this gene should have the opposite effect, which was observed by higher numbers of the bacteria in Δ*spnT* clumps. Similarly, overexpression had an insignificant negative effect on adhesion when induced with L-arabinose in the pBAD33 system in agreement with the previously reported study (60). In conclusion, the role of this gene points toward the regulator function, whereby it contributes to the regulation of cell division indirectly affecting adhesion.

Osmotic shock tolerance protein known as YggT is located in the cytoplasmic membrane and possibly forms transmembrane helices (61). The role of YggT in adhesion has not been observed or studied before. It has been reported that *yggT* is one of the genes responsible for phage shock protein (Psp) response in *Yersinia enterocolitica* and *E. coli* (62). *yggT* induced *pspA* expression, but the underlying mechanisms remain elusive. The Psp system, which responds to extracytoplasmic stress, is enhanced by the overexpression of *yggT*. The gene is also associated with signaling upon extracytoplasmic stress. *yggT* has been implicated in antibiotic resistance in *Salmonella enterica,* where *yggT*-deleted D14 mutant showed a decrease in streptomycin resistance (fourfold). Furthermore, complementation and the overexpression of *yggT* in *S. enterica* ATCC 14028 increased the streptomycin resistance (63). In this study, Δ*yggT* in the gentamicin-stressed adhesion assay as well as the gentamicin MIC assay exhibited the highest sensitivity compared to the wild type and other mutants. This can be explained by the fact that streptomycin and gentamicin are both aminoglycoside antibiotics (64). In the case of ampicillin, Δ*yggT* did not significantly alter antibiotic resistance in *S. enterica,* which is in line with our results, where ampicillin stress in adhesion and MIC assay did not significantly vary between wild type and Δ*yggT* (63). In conclusion, *yggT-*expressing membrane protein is implicated in adhesion as well as gentamicin susceptibility.

The study opens new areas of research in terms of other genes whose function still needs to be elucidated, as well as other phenotypes of *E. coli* adhesion to various cell lines, organoids, and ultimately in animal models. As it focuses on one aspect, i.e., the adhesion phenotype for antibiotic tolerances, other possibilities have not been taken into account and should also be explored, whereby bacterial physiology, such as reduced permeability and increased efflux (65), host cell invasion (66), and processes associated with the development and maintenance of cellular adhesion and clumping, may also be a contributing factor in sensitivity to the antibiotics. The differences in growth rates in the biofilm-like clumps in comparison to planktonic conditions can also affect the efficiency of antibiotic action, whereby some antibiotics exhibit strong growth rate dependence and slow growth is often associated with decreased susceptibility (67). It has been previously known that bacterial cells embedded in biofilms are extremely difficult to eliminate through antibiotics, as drug penetration is drastically reduced, and the metabolic activity of the bacterial cells is slowed down (68, 69). In terms of future perspectives, first, proteomics data from this study point toward multiple hypothetical and putative proteins whose function and role require further investigation. Second, the study will act as a foundation for testing the hypothesis that various adhesion phenotypes exhibited by pathogenic *E. coli* allow them to survive or tolerate antibiotics and other stresses that offer an advantage and enable effective pathogenesis.

## Data Availability

Whole genome sequence for strain 4972 is freely available from the GenBank database under accession number GCA_018429805.1 (13). RNA sequences of adhered and unattached bacterial strain 4972 are available in the NCBI BioProject database under accession number PRJNA868552. The whole genome sequences of all the deletion mutants for this study are available in the NCBI BioProject database under accession number PRJNA880721. All LC-MS/MS data files and MaxQuant output files have been deposited to the ProteomeXchange Consortium via the PRIDE partner repository with the data set identifier PXD042731.

## SUPPLEMENTAL MATERIAL

Supplemental material spectrum.02606-23-s0001.pdf Tables S1 to S5; Figures S1 to S6.

ASM does not own the copyrights to Supplemental Material that may be linked to, or accessed through, an article. The authors have granted ASM a non-exclusive, world-wide license to publish the Supplemental Material files. Please contact the corresponding author directly for reuse.
