# Supplementary material for: Characterization of clumpy adhesion of Escherichia coli to human cells and associated factors influencing antibiotic sensitivity
Source: Microbiol Spectr. 2024 Mar 26;12(5):e02606-23. doi: 10.1128/spectrum.02606-23 (PMC11064533; doi:10.1128/spectrum.02606-23)
Supplement: Supplemental material — Tables S1 to S5; Figures S1 to S6. [file spectrum.02606-23-s0001.pdf]

TABLE S1 Strains and Plasmids used in this study.

**Strains**	**Source**
Escherichia coli 4972	Isolated from human
Escherichia coli XL1Blue	Agilent Technologies
pKD4	Dr. Karsten Tedin, FU, Berlin, Germany
pKD46	Dr. Karsten Tedin, FU, Berlin, Germany
pCP20	Dr. Karsten Tedin, FU, Berlin, Germany
pBAD33	LGC Standard GmbH, Wesel, Germany
pFPV25.1Kan	Kolenda et. al., (1)

TABLE S2 List of strains for the comparative analysis of phylogenomic placement, phylogroup and pathotypes

**Strain ID**	**Origin/Pathotype**	**Host**	**Species/Phylogenetic group**	**Accession number**
IAI18	Urine/ExPEC	Human	*E. coli*/ C	ERX2014824
NILS24	Miscellaneous/ExPEC	Human	*E. coli*/ B1	ERX2014900
B49	Faeces/Commensal	Bird	Clade IV	ERX248048
ROAR19	Faeces/Commensal	Non-human mammal	Clade II	ERZ1458817
ROAR189	Faeces/Commensal	Non-human mammal	*E. coli*/ H	ERZ1458821
ROAR344	Faeces/Commensal	Human	*E. fergusonii*	ERZ1458823
ROAR430	Faeces/Commensal	Human	*E. coli*/ H	ERZ1458824
A03486	Faeces/Commensal	Non-human mammal	*E. coli*/ B2	ERZ1458830
B691	Faeces/Commensal	Bird	*E. fergusonii*	ERZ1458831
H442	Faeces/Commensal	Human	Clade I	ERZ1458832
DAECT14	Faeces/Commensal	Human	*E. coli*/ F	ERZ1458834
QUC093	Blood/ExPEC	Non-human mammal	*E. coli*/ F	ERZ1458835
CFT073	Urine/ExPEC	Human	*E. coli*/ B2	GCA_000007445.1
SE15	Faeces/Commensal	Human	*E. coli*/ B2	GCA_000010485.1
12009	Faeces/InPEC (EHEC)	Human	*E. coli*/ B1	GCA_000010745.1
536	Urine/ExPEC	Human	*E. coli*/ B2	GCA_000013305.1
E24377A	Faeces/InPEC (ETEC)	Human	*E. coli*/ B1	GCA_000017745.1

HS	Faeces/Commensal	Human	*E. coli*/ A	GCA_000017765.1
SMS-3-5	Water	Environment	*E. coli*/ F	GCA_000019645.1
ATCC35469T	Faeces/Unknown	Human	*E. fergusonii*	GCA_000026225.1
55989	Faeces/InPEC (EAEC)	Human	*E. coli*/ B1	GCA_000026245.1
S88	Cerebrospinal fluid/ExPEC	Human	*E. coli*/ B2	GCA_000026285.2
ED1a	Faeces/Commensal	Human	*E. coli*/ B2	GCA_000026305.1
UMN026	Urine/ExPEC	Human	*E. coli*/ D	GCA_000026325.2
IAI39	Urine/ExPEC	Human	*E. coli*/ F	GCA_000026345.1
E2348/69	Faeces/InPEC (EPEC)	Human	*E. coli*/ B2	GCA_000026545.1
042	Faeces/InPEC (EAEC)	Human	*E. coli*/ D	GCA_000027125.1
11368	Faeces/InPEC (EHEC)	Human	*E. coli*/ B1	GCA_000091005.1
E110019	Faeces/InPEC (EPEC)	Human	*E. coli*/ B1	GCA_000167875.2
53638	Faeces/InPEC (EIEC)	Human	*E. coli*/ A	GCA_000167915.2
101-1	Faeces/InPEC (EAEC)	Human	*E. coli*/ A	GCA_000168095.1
H299	Faeces/Commensal	Human	*E. coli*/ G	GCA_000176695.2
B253	Faeces/Commensal	Bird	*E. fergusonii*	GCA_000190495.1
TW09231	Water	Environment	Clade III	GCA_000208465.2
H10407	Faeces/InPEC (ETEC)	Human	*E. coli*/ A	GCA_000210475.1
NA114	Urine/ExPEC	Human	*E. coli*/ B2	GCA_000214765.3
LF82	Faeces/InPEC (AIEC)	Human	*E. coli*/ B2	GCA_000284495.1
APECO78	Lung/ExPEC	Bird	*E. coli*/ C	GCA_000332755.1
B7A	Faeces/InPEC (ETEC)	Human	*E. coli*/ B1	GCA_000725265.1
4608-58	Faeces/InPEC (EIEC)	Human	*E. coli*/ E	GCA_000805835.1
94389	Faeces/Unknown	Human	*E. albertii*/ Group 2	GCA_001514625.1
HIPH08472	Faeces/InPEC	Human	*E. albertii*/ Group 5	GCA_001514985.1
B1147	Faeces/Commensal	Bird	Clade II	GCA_001660175.1
ECOR31	Faeces/Commensal	Non-human mammal	*E. coli*/ E	GCA_001865905.1
E1118	Water	Environment	Clade V	GCA_002109985.1
H223	Urine/ExPEC	Human	*E. coli*/ B2	GCA_002110555.1
ECOR60	Urine/ExPEC	Human	*E. coli*/ B2	GCA_002189835.1
ECOR63	Faeces/Commensal	Human	*E. coli*/ B2	GCA_002189905.1
ECOR64	Urine/ExPEC	Human	*E. coli*/ B2	GCA_002189945.1
ECOR01	Faeces/Commensal	Human	*E. coli*/ A	GCA_002190105.1
ECOR24	Faeces/Commensal	Human	*E. coli*/ A	GCA_002190595.1
ECOR42	Faeces/Commensal	Human	*E. coli*/ E	GCA_002190935.1
ECOR49	Faeces/Commensal	Human	*E. coli*/ D	GCA_002190975.1
SAKAI	Faeces/InPEC (EHEC)	Human	*E. coli*/ E	GCA_003028755.1
RDEx444	Faeces/ExPEC-InPEC (EHEC)	Human	*E. coli*/ A	GCA_003123505.1
4051-6	Faeces/InPEC	Human	*E. albertii*/ Group 3	GCA_012532675.1
S286	Cerebrospinal fluid/ExPEC	Human	*E. coli*/ C	GCA_013363015.1
CIP61.11	Faeces/Commensal	Human	*E. coli*/ A	GCA_900236115.1
FN-B26	Faeces/Commensal	Human	*E. coli*/ B2	GCA_902505495.1
H1-003-0072	Blood/ExPEC	Human	*E. coli*/ D	GCA_902709615.2
H1-006-0003	Blood/ExPEC	Human	*E. coli*/ C	GCA_902711215.2
H1-006-0025	Blood/ExPEC	Human	*E. coli*/ B2	GCA_902711405.2
H176	Faeces/Commensal	Human	*E. coli*/ B2	GCA_902842355.1
2H-327-20	Faeces/Commensal	Human	*E. coli*/ G	GCA_902849165.1
EC03-127	Faeces/InPEC	Human	*E. albertii*/ Group 4	GCA_001514885.1
K7756	Faeces/InPEC	Human	*E. albertii*/ Group 1	GCA_001515045.1

TABLE S3 Details of samples and their corresponding cell lines for the RNA-sequencing.

Sample number	Sample ID	Type	Medium	Cell line	Cell line Source
1	S1	supernatant	RPMI 10 % FBS	5637	Human
2	C1	adhering/clumps	RPMI 10 % FBS	5637	Human
3	S2	supernatant	DMEM 10 % FBS	PK15	Pig
4	C2	adhering/clumps	DMEM 10 % FBS	PK15	Pig
5	S3	supernatant	DMEM 10 % FBS	CaCo-2	Human
6	C3	adhering/clumps	DMEM 10 % FBS	CaCo-2	Human
7	DMEM	control	DMEM 10 % FBS	-	
8	LB	control	LB	-	

TABLE S4 List of all primers.

ID	Name	Length (bp)	Primer sequence (5'-3')	Reference
O2921	fwd-flgH-DW-*E.coli*	93	ATGCAAAAAAACGCTGCGCATAC TTATGCTATTTCCAGCTTGTTGGT GCTGTGTAGGCTGGAGCTGCTTC	This Study
O2922	Rev-flgH-DW-*E.coli*	93	TTACATTGGCGACAGGTTAAGGAA GAAACGCTGCAACCAGCCCATATT TTCATATGAATATCCTCCTTAG	This Study
O2962	fwd-spy-DW-*E.coli*	70	ATGCGTAAATTAACTGCACTGTTT GTTGCCTCTACCCTGGCTCTTGGC GCGTGTAGGCTGGAGCTGCTTC	This Study
O2963	rev-spy-DW-*E.coli*	70	TTATTCAGCAGTTGCAGGCATTTT ACCTTTTGCCGCTGGACGTTCTGT CACATATGAATATCCTCCTTAG	This Study
O2988	attB1YggtA-fwd	49	GGGGACAAGTTTGTACAAAAAAGCA GGCTGGCAGCGTCAATTTGGTGAA	This Study
O2989	YggtPmeI-1-rev	58	CCAGACCGTCATCATTAACTGTTACG GCACGTTTAAACACGAACCATCGTGC TACCTG	This Study
O2990	YggtPmeI-2-fwd	58	CTCTAAAAAATAAGGAATTAAAGGA ACGCCGTTTAAACCGGTCTGGTTTTA CGGCTCT	This Study
O2991	attB2Yggt-rev	49	GGGGACCACTTTGTACAAGAAAGCTG GGTGTCGTCCGGTACGTCTTTCA	This Study
O3000	SeqLA1 – fw	25	CTCTCGCGTTAACGCTAGCATGGAT	(2)
O3001	SeqLB – rev	24	GTAACATCAGAGATTTTGAGACAC	(2)
O3004	AttB1FimH-fwd	49	GGGGACAAGTTTGTACAAAAAAGCA GGCTGCGTGTTGAAGCAAACTATC	This Study
O3005	FimHPmeI-1-rev	58	CCTACAGCTGAACCCGAAGAGATGA TTGTAGTTTAAACAGAAATCACAGG GCATTGCT	This Study
O3006	FimHPmeI-2-fwd	61	TACCTGCATTAGCAATGCCCTGTGAT TTCTGTTTAAACTACAATCATCTCTT CGGGTTCAG	This Study
O3007	AttB2FimhH-rev	49	GGGGACCACTTTGTACAAGAAAGCTG GGTCACGGCTACCTTCACTCTTG	This Study
O3021	flgHXmaI-for	39	CACCCGGGAGGAGGACAGCTATGCAAAAAAACGCT GCGC	This Study
O3022	flgHXbaI-rev	32	ACATTCTAGATTACATTGGCGACAGGTTAAGG	This Study
O3039	AttB1PilV_F	51	GGGGACAAGTTTGTACAAAAAAGCAGGCT CCGGATTGACAAATACTGTTGC	This Study
O3040	PilVPmeI-1_R	62	TTTCAATTATAGTAAGTAAGAGTTAATCTC GTTTAAACTTTTATATTCACGTATTAATTAAT	This Study
O3041	PilVPmeI-2_F	57	TTGATAATTAATTAATACGTGAATATAAAA GTTTAAACGAGATTAACTCTTACTTAC	This Study
O3042	AttB2PilV_R	51	GGGGACCACTTTGTACAAGAAAGCTGGGTA GTCAGTTTACGAATACGTTCC	This Study
O3046	AttB1ffp_F	49	GGGGACAAGTTTGTACAAAAAAGCAGGCTC ATTTCCGACTGTCCGATAG	This Study
O3047	ffpPmeI-1_R	58	AATTTAAATACTATTTTACAGGTAAATTTAG TTTAAACTCTATCTCCTCCACTTTAAC	This Study
O3048	ffpPmeI-2_F	58	CTCCCCCAAAGTTAAAGTGGAGGAGATAGA GTTTAAACTAAATTTACCTGTAAAATAG	This Study
O3049	AttB2ffp_R	49	GGGGACCACTTTGTACAAGAAAGCTGGGTT CTTTTATTGTGCAAAGTGC	This Study

O3065	AttB1SpnT_F	49	GGGGACAAGTTTGTACAAAAAAGCAGGCTCCGGCATAACTTTTTTGAAG	This Study
O3066	SpnTPmeI-1_R	56	CGGCCTTTCAACTTCATGCTTAATTACAATGTTTAAACCATCACATCCTGATAATG	This Study
O3067	SpnTPmeI-2_F	60	GCGTCTACCAAACATTATCAGGATGTGATGGTTTAAACATTGTAATTAAGCATGAAGTTG	This Study
O3068	AttB2SpnT_R	49	GGGGACCACTTTGTACAAGAAAGCTGGGTTTCGCTCGCTGGTCAGAAAT	This Study
O3196	ffpKpnIFor	42	CAGGTACCAGGAGGACAGCTATGACCTGTGGTTTTGAAGATC	This Study
O3197	ffpSalIRev	29	ACATGTCGACTTAGTTGTACACAACCACG	This Study
O3198	pilVKpnIFor	38	CAGGTACCAGGAGGACAGCTATGGCGATGAAAATGAAA	This Study
O3199	pilVSalIRev	32	ACATGTCGACTTAATTTACAATTCTTATTGCA	This Study
O3200	spnTKpnIFor	42	CAGGTACCAGGAGGACAGCTATGGCTAAACTGAACAAGAAAC	This Study
O3201	spnTSalIRev	34	ACATGTCGACCTATGCTGAATATGATACTGAATC	This Study
O3202	yggTKpnIFor	41	CAGGTACCAGGAGGACAGCTATGAATACGTTGACTTTCCTG	This Study
O3203	yggTSalIRev	28	ACATGTCGACAACGCCATCCACAGCCCC	This Study

TABLE S5 Decisions used for pathotyping and RefSeq genomes assigned to each pathotype.

Pathotype	Genes present	Genes absent	No. of Genomes
tEPEC	*eae, bfp*	*stx1a, stx1b, stx2a, stx2b*	115
aEPEC	*eae*	*bfp, stx1a, stx1b, stx2a, stx2b*	612
STEC	*stx1a* and *stx1b* or *stx2a* and *stx2b*	*aatA, eae, ial, ipaH, eltA, eltB, sta1, sta2, stb, aggR, afaA, afaE-I, afaE-III, daaE, draE, sat, vat*	488
EHEC	*stx1a, stx1b, stx2a, stx2b, eae* or *stx1a, stx1b, eae* or *stx2a, stx2b, eae*		1725
EAEC	*aggR, aatA*		163
ETEC	One of: *eltA, eltB, sta1, sta2*	*aggR*	551
EIEC	*ial* or *ipaH*	*stx1a, stx1b, stx2a, stx2b*	30
DAEC	*afaA, sat,* one of: *afaE-I, afaE-III, daaE, draE*		229
UPEC	*fyuA, fimH,* two of: *chuA, yfcV, vat*		2598
NMEC	*sitA, vat, neuC, iucC, neuA*		11
Nonpathogenic	possessing only one of the following genes: *fyuA, iucC, neuC, sitA, yfcV* and nothing else	*aap, aatA, aatC, aatP, afaA, afaB, afaC, afaD, afaE-I, afaE-III, aggR, bfpA, daaE, draE, eae, eltA, eltB, ial, ipaH, pet, sat, sta1, sta2, stb, stx1a, stx1b, stx2a, stx2b, vat*	5265

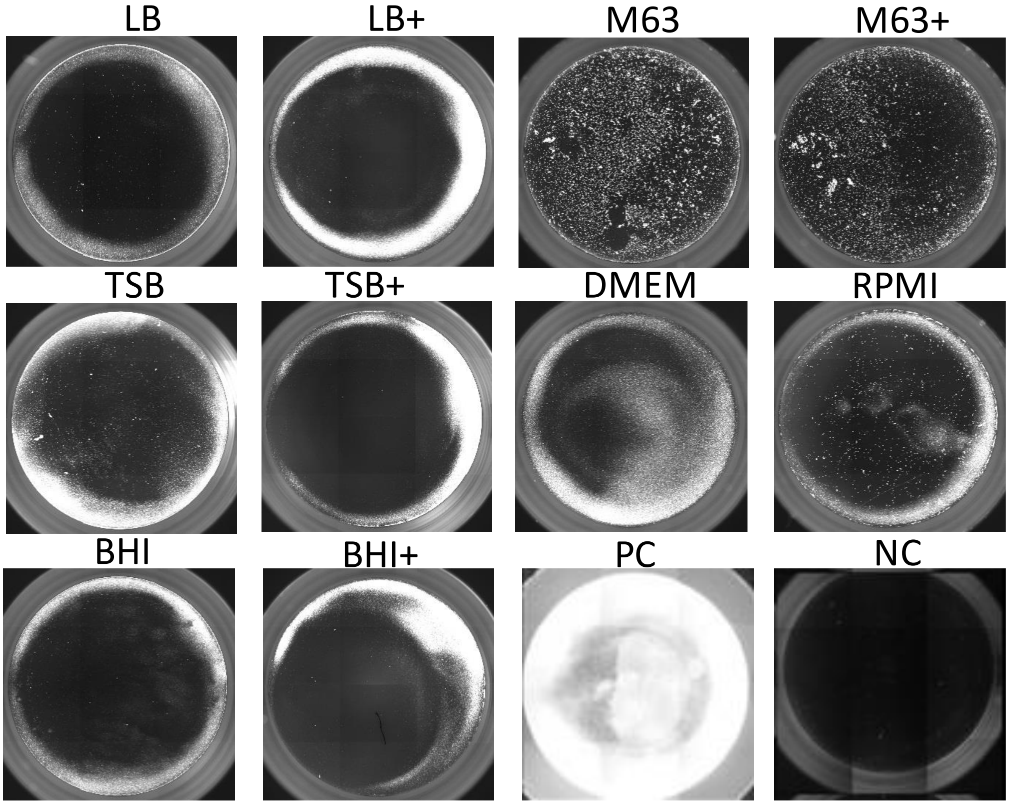

FIG S1 Biofilm formation.

Single-well overview images of strain 4972 showing biofilm formation in different media. + indicates addition of glucose. PC is biofilm formation by the positive control strain of *E. coli* MG1655 F′Tet Δ*traD* and NC is the negative control i.e., media without bacteria. Fluorescence images were captured after 48 h of biofilm formation in respective media and staining with SYTO 9.

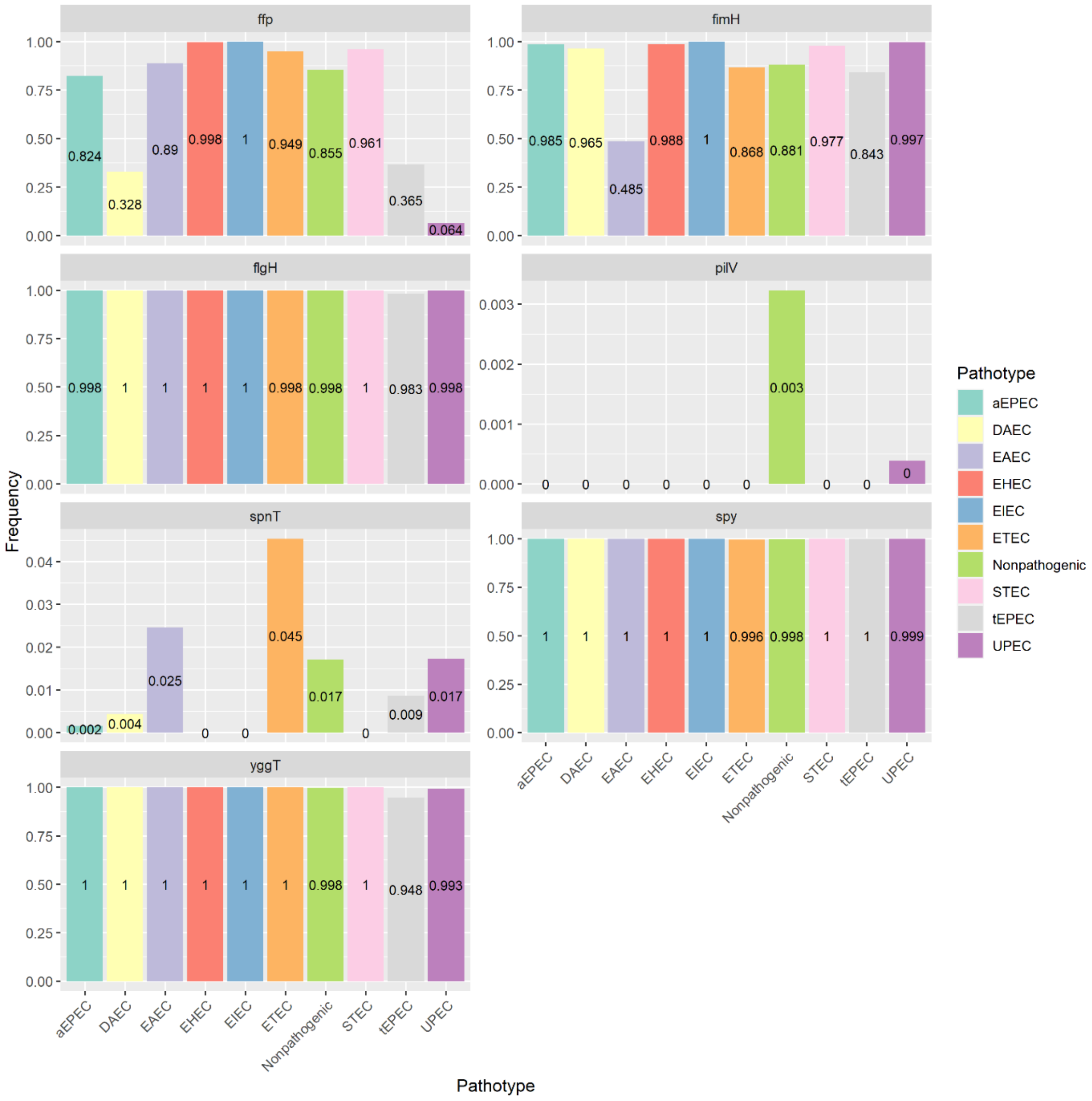

FIG S2 Prevalence of targeted genes in *E. coli* pathotypes.

Gene prevalence by using Abricate and *E. coli* genomes downloaded from RefSeq database across nine pathotypes and nonpathogenic *E. coli* are shown in each graph. x-axis shows the pathotype and y-axis shows the prevalence with the coverage of 95 %.

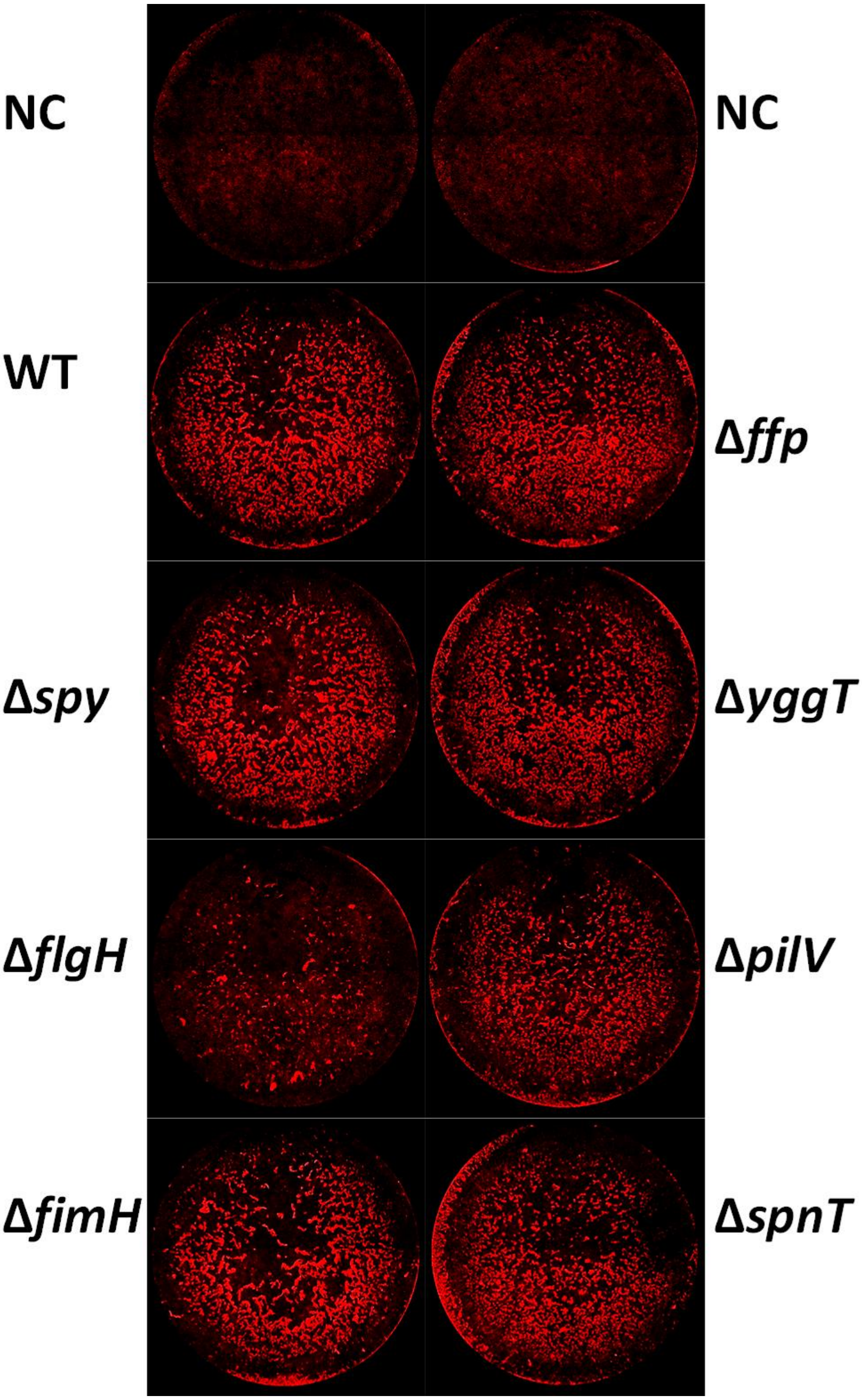

FIG S3. Clump formation by wild-type strain and deletion mutants on 5637 cell line after 4 h incubation.

The fluorescent staining was done by Propidium iodide (PI) and visualized by Aklides system. NC is PI stained 5637 cell line only as negative control

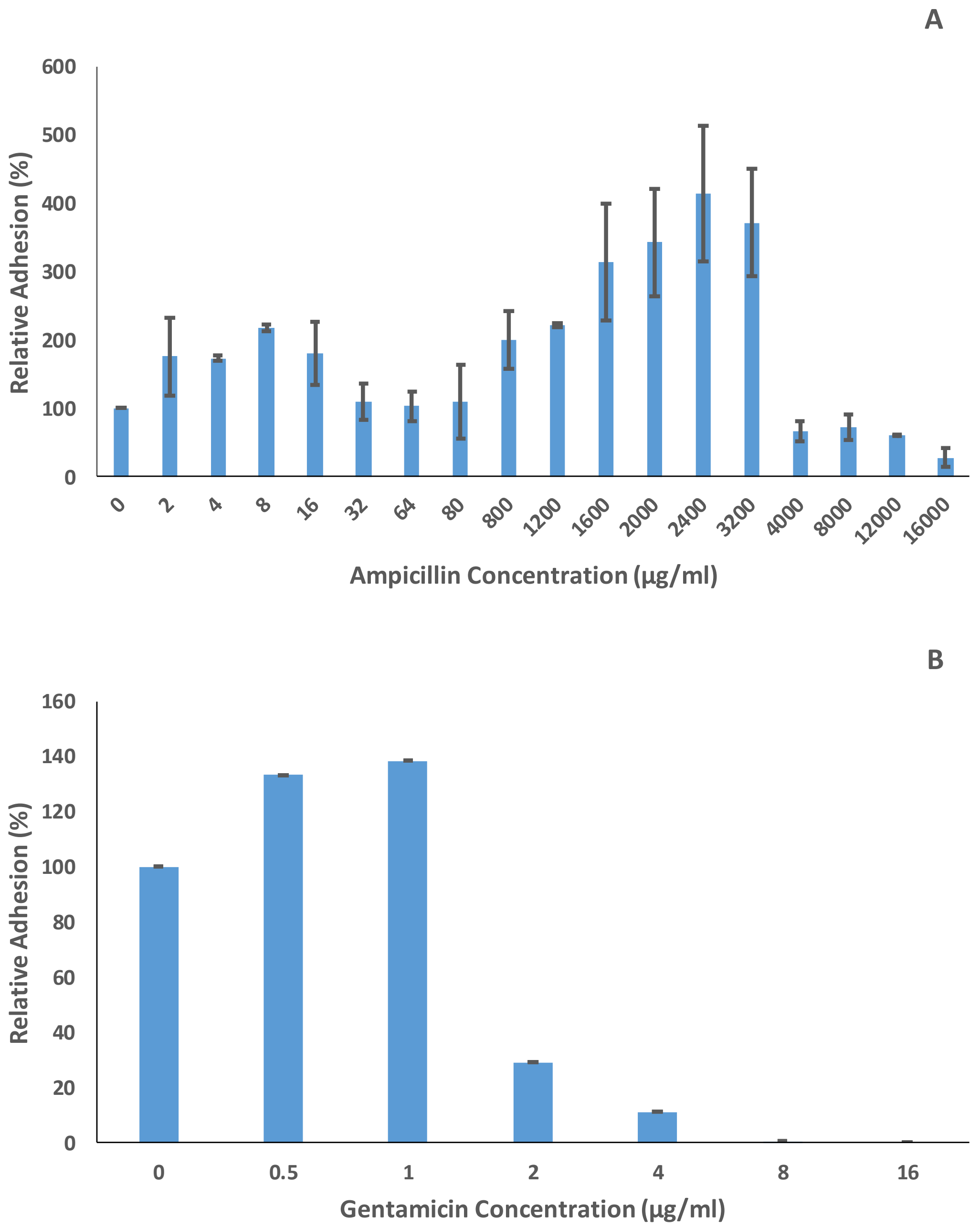

FIG S4 Results of antibiotics dose ranging experiments.

A: Ampicillin concentrations used to stress the clumpy structure of the wild type ranging from 0 to 16,000 µg/ml. B: Gentamicin concentration used to stress the clumpy structure of wild-type strain ranging from 0-16 µg/ml. Each concentration was checked in triplicate in one experiment and relative adhesion percentage with respect to adhesion without antibiotic exposure i.e., 0 µg/ml (median value normalized to 100%) is shown on y-axis and antibiotic concentrations on x-axis. The data comprises of median values and median absolute deviation (MAD).

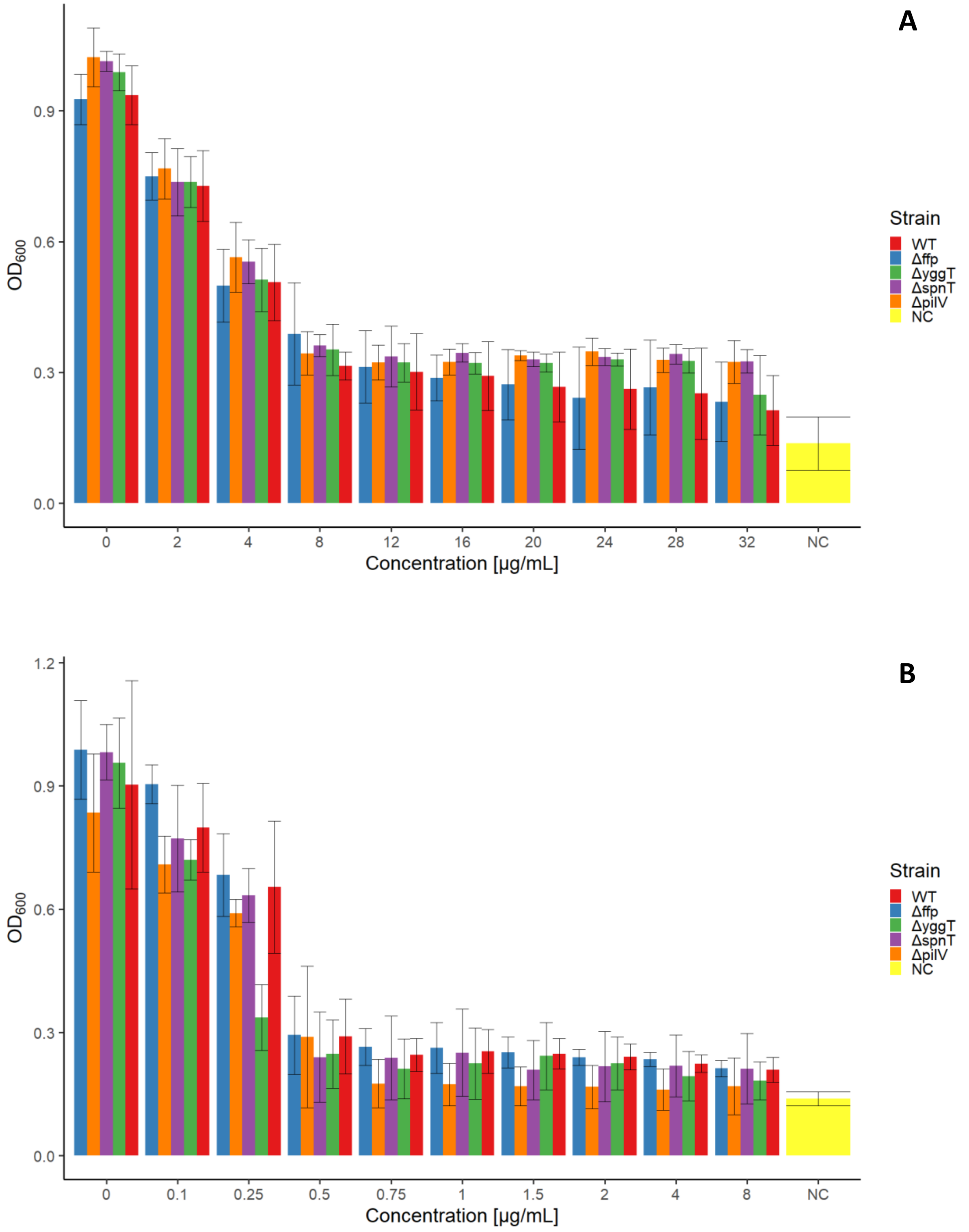

FIG S5. MIC assay

A: MIC assay of wild-type strain 4972 and the unannotated gene deletion mutants with a range of ampicillin concentrations i.e., 0-32 µg/ml. B: MIC assay of wild-type strain 4972 and the unannotated with a range of gentamicin concentrations i.e., 0-8 µg/ml. NC refers to as negative control. The data comprises of median values and median absolute deviation (MAD) of five separate experiments in triplicate. The concentrations of the respective antibiotics are shown on x-axis and OD_{600} is show on the y-axis.

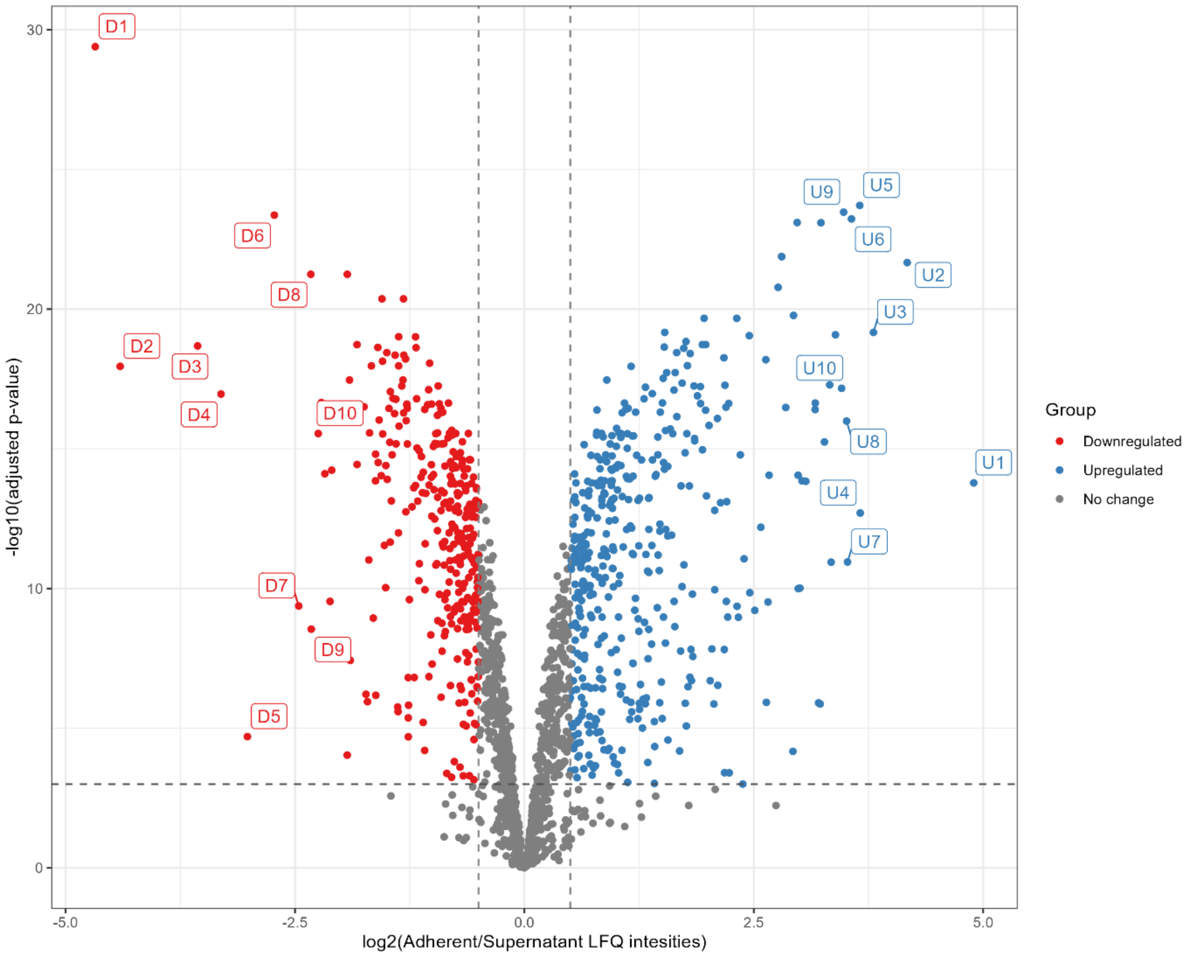

FIG S6. Volcano plot of most differentially expressed proteins between adhered bacteria and planktonic bacteria in the supernatant

The most upregulated proteins (blue dots) are U1: Ribosome modulation factor, U2: Cytochrome bd-I ubiquinol oxidase subunit 2, U3: Phosphoenolpyruvate-dependent phosphotransferase system, U4: Hypothetical protein, U5: Penicillin-binding protein 1B, U6: HTH-type transcriptional regulator MalT, U7: Polyphosphate kinase, U8: D-galactose-binding periplasmic protein, U9: Hypothetical protein and U10: Hypothetical protein. The top ten down regulated proteins (red dots) are D1: Hypothetical protein, D2: tRNA-2-methylthio-N(6)-dimethylallyladenosine synthase, D3: Protein YobA, D4: Cold shock-like protein CspG, D5: putative protein, D6: Hypothetical protein, D7: Hypothetical protein, D8: Hypothetical protein, D9: Hypothetical protein and D10: Hypothetical protein.

References

1. Kolenda R, Burdukiewicz M, Wimonć M, Aleksandrowicz A, Ali A, Szabo I, Tedin K, Bartholdson Scott J, Pickard D, Schierack P. 2021. Identification of natural mutations responsible for altered infection phenotypes of *Salmonella enterica* clinical isolates by using cell line infection screens. Appl Environ Microbiol 87:e02177-20.

2. Derous V, Deboeck F, Hernalsteens J-P, De Greve H. 2011. Reproducible gene targeting in recalcitrant *Escherichia coli* isolates. BMC Res Notes 4:213–213.
